# Palladium-catalyzed C–N and C–O bond formation of N-substituted 4-bromo-7-azaindoles with amides, amines, amino acid esters and phenols

**DOI:** 10.3762/bjoc.8.227

**Published:** 2012-11-19

**Authors:** Rajendra Surasani, Dipak Kalita, A V Dhanunjaya Rao, K B Chandrasekhar

**Affiliations:** 1 Custom Pharmaceutical Services, Dr. Reddy’s Laboratories Ltd., Miyapur, Hyderabad 50049, India; 2 Department of Chemistry, Institute of Science and Technology, JNTU University of Anantapur, Anantapur 515002, India

**Keywords:** 7-azaindole, C–N bond, C–O bond, ligand, palladium catalyst

## Abstract

Simple and efficient procedures for palladium-catalyzed cross-coupling reactions of N-substituted 4-bromo-7-azaindole (1*H*-pyrrole[2,3-*b*]pyridine), with amides, amines, amino acid esters and phenols through C–N and C–O bond formation have been developed. The C–N cross-coupling reaction of amides, amines and amino acid esters takes place rapidly by using the combination of Xantphos, Cs_2_CO_3_, dioxane and palladium catalyst precursors Pd(OAc)_2_/Pd_2_(dba)_3_. The combination of Pd(OAc)_2_, Xantphos, K_2_CO_3_ and dioxane was found to be crucial for the C–O cross-coupling reaction. This is the first report on coupling of amides, amino acid esters and phenols with N-protected 4-bromo-7-azaindole derivatives.

## Introduction

Palladium-catalyzed C–N and C–O bond-forming reactions between 4-substituted 7-azaindoles and amides, amines, amino acid esters or phenols have recently gained popularity among the scientific community for different drug-discovery and -development programs. Particularly, several 7-azaindoles (1*H*-pyrrole[2,3-*b*]pyridine) [1–4], including 4-substituted compounds [5–8] have found applications in various therapeutic areas. Despite their utility in various drug-development programs in academic research and the pharmaceutical industry, methods for the synthesis of this class of compounds and functionalization of 7-azaindole scaffolds remain limited [9]. Although the literature enumerates various methods for the synthesis of substituted azaindole motifs, they are limited to N-1, C-2 or C-3 functionalized structures [5–8,10]. Furthermore, regioselective C–O-bond-forming reactions are interesting in organic synthesis due to the presence of these bonds in numerous natural products, biological compounds, pharmaceuticals, fragrances, cosmetics and polymers [11–17]. Among others Buchwald, Hartwing and co-workers followed by many other groups during the past decade reported the metal-catalyzed formation of carbon–heteroatom bonds [18–30]. Most of the literature reports are limited to aryl halides and indoles only. Clearly, each of these protocols has its own virtues; however, limitations still exist with respect to substrate scope, reagents and solvents, etc. Thus, palladium-catalyzed intra- and intermolecular cross-coupling reactions of azaindoles with amides, amines, amino acid esters or phenols offer an interesting complementary method for the synthesis of C–N and C–O bonds under comparably mild conditions. It is important to note that, in contrast to well-established palladium-catalyzed coupling reactions of indole with amines, alcohols and phenols [5,7,31–36], very few studies on the formation of C–N and C–O bond formation over 7-azaindole have been performed [37–39]. On the other hand, the chemistry of 4-bromo-7-azaindole has not been explored in depth until today.

**Figure 1:**
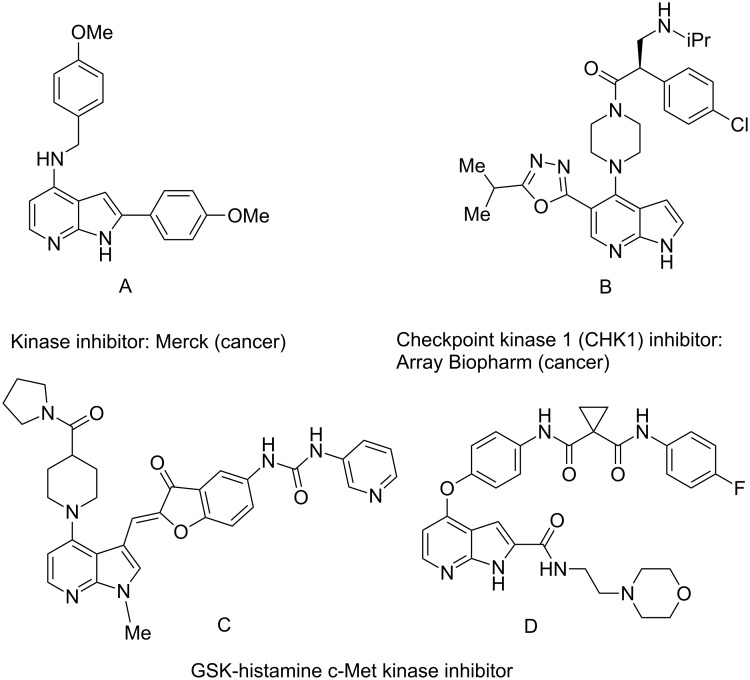
Representative drug candidates of amino-azaindole and phenyl-azaindole containing motifs.

Amino and phenyl-substituted 7-azaindole scaffolds appear in various pharmaceutically important molecules (Figure 1), which are very challenging and lengthy to prepare by the traditional methods [40–41]. In general, nucleophilic aromatic substitution (S_N_Ar) reaction of a halo-precursor of 7-azaindole with a large excess of amine counterpart under high reaction temperatures, preferably under heating to more than 180 °C or through the use of microwave irradiation, results in the amino-7-azaindole in moderate to low yield [5,7]. Primary alkylamines or anilines under similar reaction conditions provided the displacement rearrangement products 4-amino-5-azaindole as the sole product [7]. Very recently Buchwald et al. [39] reported a palladium-catalyzed amination of unprotected halo-7-azaindoles using biarylphosphine ligands (DavePhos), palladium precatalyst (RuPhos)-based reagents, and LiHDMS as a base. However, inconsistency of the results was observed when the reactions were carried out on a large scale. As part of continuing efforts in our laboratory [42–46] toward the development of new and improved methods in organic synthesis, we became interested in the possibility of developing an efficient palladium-mediated coupling of amides, amines, amino acid esters and phenols with N-protected 7-azaindole derivatives for one of our medicinally important drug-development programs.

**Scheme 1:**
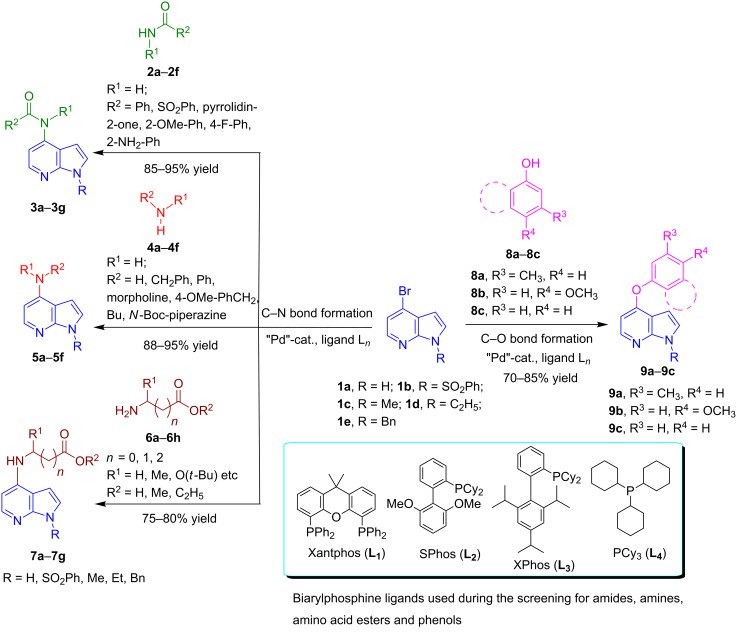
Cross coupling of 4-bromo-7-azaindole with amides, amines, amino acid esters and phenols.

We herein report on Pd-catalyzed coupling reactions of N-protected 4-bromo-7-azaindoles with amides, amines, amino acid esters and phenols (Scheme 1), to yield new important intermediates for one of our medicinal chemistry programs. To the best of our knowledge this is the first report on intermolecular coupling of 4-bromo-7-azaindole with amides, amino acid esters and phenols.

## Results and Discussion

4-Bromo-7-azaindole derivative **1** was prepared from 7-azaindole by the literature procedure [47]. In most of the cases we have used the N-protection of 4-bromo-7-azaindole. It is worth mentioning that 7-azaindole (1*H*-pyrrole[2,3-*b*]pyridine), has a [4.3]-bicyclic indene skeleton with a fused electron-rich pyrrole ring and an electron-deficient pyridine ring. The p*K*a value of 7-azaindole is ~4.9, and it undergoes self-association through hydrogen-bonding to form a dimer in solution and phototautomerizes by an excited-state double-proton-transfer (ESDPT) process [1,48]. In the presence of copper or palladium catalysts azaindole undergoes arylation of the heterocyclic N–H nitrogen [49–50]. To find a suitable condition for amide coupling with 7-azaindole derivatives, various biaryl/alkyl phosphine ligands, palladium catalysts, bases and reaction times, etc., were screened by using electron-deficient N-protected 4-bromo-7-azaindoles **1** as substrates (Table 1). *N*-Benzyl-4-bromo-7-azainole (1-benzyl-4-bromo-1*H*-pyrrolo[2,3-*b*]pyridine, **1e**) and benzamide (**2a**) were chosen as model substrates to find the suitable palladium-mediated coupling of amides with N-protected 4-bromo-7-azaindole **1**. After extensive screening, we found that the combination of Pd(OAc)_2_, Xantphos (**L_1_**) as a ligand [51–53], Cs_2_CO_3_ as a base and dioxane as a solvent provided the most successful result (Table 1, entry 1). The reaction temperature was maintained at ~100 °C in all cases. Switching the Pd source to Pd_2_(dba)_3_ resulted in a slight decrease in yield (Table 1, entry 2). Other available ligands, e.g., SPhos (**L_2_**) and XPhos (**L_3_**) provided lower yields when Pd_2_(dba)_3_ was used as a catalyst, even after longer reaction time (Table 1, entries 3 and 4). By using Pd(OAc)_2_ as a catalyst and SPhos (**L_2_**) and XPhos (**L_3_**) as a bidentate ligand, low to moderate yields were obtained (Table 1, entries 6 and 7). When tertiary ligand PCy_3_ (**L_4_**) was used as a ligand for the cross-coupling reaction no product formation was observed (Table, entry 5). Cross-coupling reaction of *N*-benzyl-4-bromo-7-azaindole (**1e**) and benzamide (**2a**) with other bases, e.g., K_2_CO_3_ and K_3_PO_4_, by using Pd(OAc)_2_ and Xantphos (**L_1_**) as a ligand provided good yield in 4 to 3 h (Table 1, entries 8 and 9). It is worth mentioning that Xantphos (**L_1_**) as a supporting ligand finds wide popularity in palladium-mediated amidation reactions by various research groups [54–56], which prompted us to evaluate the process further, with various substrate scopes.

**Table 1:** Reaction optimization for coupling of **1e** with benzamide (**2a**) under various conditions^a^.

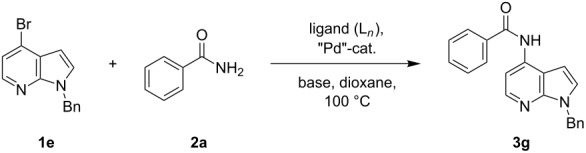					

Entry	Pd catalyst (5 mol %)	L*_n_*	Base	Time (h)	Yield (%)^b^

1	Pd(OAc)_2_^c^	**L_1_**	Cs_2_CO_3_	2	95^d^
2	Pd_2_(dba)_3_	**L_1_**	Cs_2_CO_3_	5	90
3	Pd_2_(dba)_3_	**L_2_**	Cs_2_CO_3_	24	40
4	Pd_2_(dba)_3_	**L_3_**	Cs_2_CO_3_	24	40
5	Pd_2_(dba)_3_	**L_4_**	Cs_2_CO_3_	24	0
6	Pd(OAc)_2_	**L_2_**	Cs_2_CO_3_	24	45
7	Pd(OAc)_2_	**L_3_**	Cs_2_CO_3_	24	61
8	Pd(OAc)_2_	**L_1_**	K_2_CO_3_	4	83
9	Pd(OAc)_2_	**L_1_**	K_3_PO_4_	3	88

^a^Reactions of 1-benzyl-4-bromo-1*H*-pyrrolo[2,3-*b*]-pyridine (**1e**) (1.0 mmol) with benzamide (**2a**) (1.2 mmol) were performed in a sealed Schlenk tube at 100 °C in dioxane (2 mL) by using Pd catalyst (5 mol %), ligand (10 mol %) and base (1.5 mmol). ^b^Yields reported are isolated yields. ^c^No reaction occurred without palladium catalyst. ^d^No reaction occurred at room temperature.

With optimized conditions in hand, we embarked on an investigation of the reaction scope by subjecting various N-protected 7-azaindoles **1** to a wide range of amides **2**. The experimental results are summarized in Table 2. The reaction did not proceed at all without N-protection (**1a**, Table 2, entry 1). When the reaction was carried out with *N*-sulfonyl-protected 4-bromo-7-azaindole **1b** only the desulfonated product (Table 2, entry 2) was obtained. It is worth mentioning that the *N*-sulfonyl protected 7-azaindole **1b** was efficiently deprotected under basic conditions in dioxane [57]. The optimized reaction conditions worked well with benzamide (**2a**) (Table 2, entry 3) and phenylsulfonamide (**2b**) (Table 2, entry 4) to obtained a good yield. A cyclic secondary amide (lactam) **2c** also reacted efficiently (Table 2, entry 5). The methodology works equally well with 2-methoxybenzamide (**2d**) (Table 2, entry 6) and 4-fluorobenzamide (**2e**) (Table 2, entry 7). We checked the selectivity of amide and amine coupling by reacting *N*-ethyl-7-bromoazaindole (**1d**) with 2-aminobenzamide (**2f**) and obtained 2-amino-*N*-(1-ethyl-1*H*-pyrrolo[2,3-*b*]pyridin-4-yl)benzamide (**3f**) in 85% yield (Table 2, entry 8). We found that amide is more reactive than amine under the reaction conditions studied. The use of Cs_2_CO_3_ as the base is advantageous because the common functional groups such as fluoro, methoxy, etc., are well tolerated. We found that the N-protection of 4-bromo-7-azaindoles **1** has a marginal effect on the reaction yield and time. The coupling of amides with N-protected 4-bromo-7-azaindoles **1** was demonstrated in multi-gram synthesis in our hands. Next we diverted our attention towards coupling of N-protected 4-bromo-7-azaindoles **1** with amines **4**.

**Table 2:** C–N-bond-formation cross coupling of N-protected 4-bromo-7-azaindoles **1** with amides **2**.

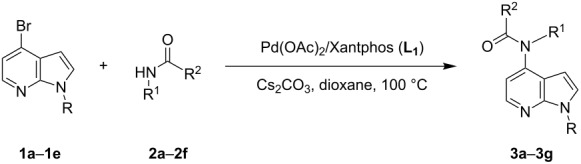					

Entry	7-Azaindole **1**	Amide **2**	Product **3**^a^	Time (h)	Yield (%)^b^

1	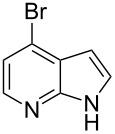 **1a**	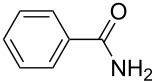 **2a**	–	24	NR^c^
2	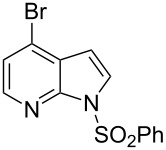 **1b**	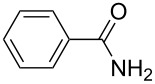 **2a**	–	5	0^d^
3	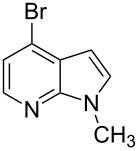 **1c**	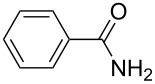 **2a**	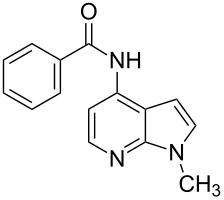 **3a**	2	95
4	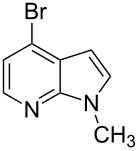 **1c**	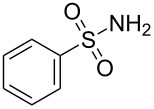 **2b**	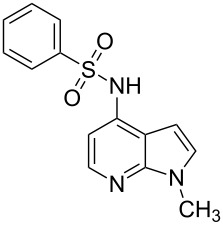 **3b**	3	85
5	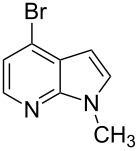 **1c**	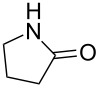 **2c**	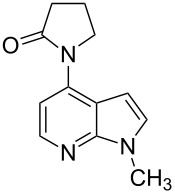 **3c**	2.5	92
6	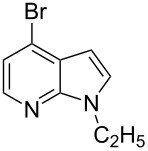 **1d**	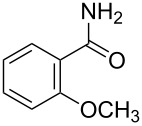 **2d**	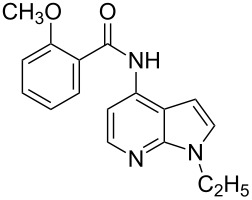 **3d**	3	91
7	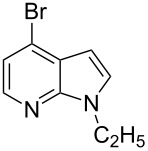 **1d**	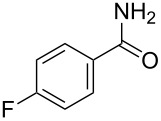 **2e**	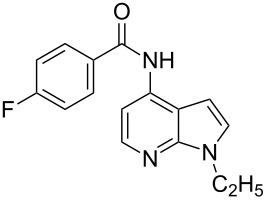 **3e**	3	89
8	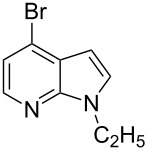 **1d**	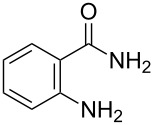 **2f**	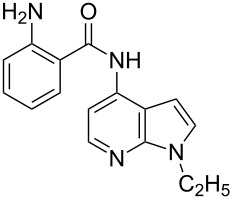 **3f**	3	85
9	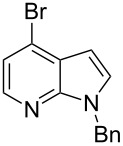 **1e**	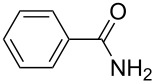 **2a**	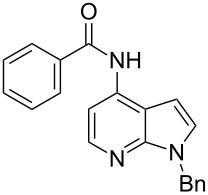 **3g**	2	95

^a^Reactions of N-protected 7-azaindoles **1** (1.0 mmol) with amides **2** (1.2 mmol) were performed in a sealed Schlenk tube at 100 °C in dioxane (2 mL) by using Pd(OAc)_2_ (5 mol %), Xantphos (10 mol %) and base (1.5 mmol). ^b^Yields reported are isolated yields. ^c^NR no reaction. ^d^Desulfonation reaction takes place.

Synthesis of 4-amino-7-azaindoles was generally achieved from the corresponding halide by S_N_Ar displacement reactions, which typically require very high temperatures, extended reaction times, and a large excess of the amine counterpart [5]. Other alternative methods employ the amino-substituted azaindole as the key intermediate, which are challenging to prepare [6]. Initially, coupling of 4-bromo-1-ethyl-1*H*-pyrrolo[2,3-*b*]pyridine (**1d**) with phenylmethanamine (**4a**) was selected as a model reaction to optimize the reaction condition of C–N-bond formation of amines. The experimental results are summarized in Table 3. After the screening of various ligands (Scheme 1), palladium catalysts, and bases (Table 1), the catalyst combination of Pd_2_(dba)_3_, Xantphos and Cs_2_CO_3_ in dioxane was found to be crucial. The cross-coupling reaction of 4-bromo-1-ethyl-1*H*-pyrrolo[2,3-*b*]pyridine (**1d**) with phenylmethanamine (**4a**) proceeded rapidly by using the combination of Pd_2_(dba)_3_, Xantphos and Cs_2_CO_3_ in dioxane at 100 °C for 1 h (Table 3, entry 1). When K_2_CO_3_ was used as base along with Pd_2_(dba)_3_ as catalyst and XantPhos (**L_1_**) as ligand, slightly lower yield (~85%) was obtained (Table 3, entry 2) in 3 h. Other ligands SPhos (**L_2_**) and XPhos (**L_3_**) with Pd_2_(dba)_3_ as catalyst provided average yields of 60 and 62%, respectively, in 6 h (Table 3, entries 3 and 4). However, the tertiary phosphine ligand PCy_3_ (**L_4_**) was ineffective in generating any product (Table 3, entries 5 and 12). Interestingly, Pd(OAc)_2_ results in poor yields of the product (Table 3, entries 6–12). Given this surprising result, we hypothesized that the amination product **5** may interfere with catalyst turnover by promoting the formation of an inactive Pd-chelate complex.

**Table 3:** Optimization of the coupling reaction of 4-bromo-1-ethyl-1*H*-pyrrolo[2,3-*b*]pyridine (**1d**) with phenylmethanamine (**4a**).^a^

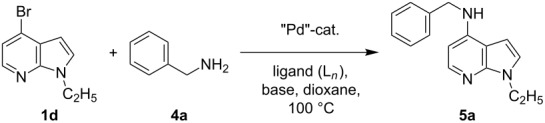					

Entry	Pd-catalyst (5 mol %)	L*_n_*	Base	Time (h)	Yield (%)^b^

1	Pd_2_(dba)_3_	**L_1_**	Cs_2_CO_3_	1	93
2	Pd_2_(dba)_3_	**L_1_**	K_2_CO_3_	3	85
3	Pd_2_(dba)_3_	**L_2_**	Cs_2_CO_3_	6	60
4	Pd_2_(dba)_3_	**L_3_**	Cs_2_CO_3_	6	62
5	Pd_2_(dba)_3_	**L_4_**	Cs_2_CO_3_	24	0
6	Pd(OAc)_2_	**L_1_**	Cs_2_CO_3_	24	20
7	Pd(OAc)_2_	**L_1_**	K_2_CO_3_	24	15
8	Pd(OAc)_2_	**L_1_**	NaO*t*-Bu	24	23
9	Pd(OAc)_2_	**L_1_**	K_3_PO_4_	24	20
10	Pd(OAc)_2_	**L_2_**	Cs_2_CO_3_	24	18
11	Pd(OAc)_2_	**L_3_**	Cs_2_CO_3_	24	17
12	Pd(OAc)_2_	**L_4_**	Cs_2_CO_3_	24	0

^a^Reactions of 1-ethyl-4-bromo-1*H*-pyrrolo[2,3-*b*]-pyridine (**1d**) (1.0 mmol) with phenylmethanamine (**4a**) (1.2 mmol) were performed in a sealed Schlenk tube at 100 °C in dioxane (2 mL) by using Pd catalyst (5 mol %), ligand (10 mol %) and base (1.5 mmol). ^b^Yields reported are isolated yield.

With a viable coupling procedure in hand, attention was turned to the generality of the process and couplings of structurally diverse nucleophilic amines. As seen from Table 4, the cross-coupling reaction of N-protected 4-bromo-7-azaindoles **1a**–**1d** with various amines **4a**–**4f** proved to be general under the optimized conditions to get the coupled products **5a**–**5f** in very good yield (88–94%) within a reasonable time of 2.5 to 3 h. The C–N-bond-forming reaction of primary aromatic amines (Table 4, entries 3, 4 and 6) proceeded smoothly under the optimized conditions to provide excellent yields of the corresponding coupling products **5a**, **5b** and **5d**, respectively. The reaction was also effective for cyclic amine morpholine (Table 4, entry 5) and Boc-protected piperazine (Table 4, entry 8). The reaction works equally well for aliphatic primary amine too (Table 4, entry 7) resulting in 90% isolated yield. There was a feeble change in yield by varying the substitution on the 7-azaindole nitrogen (N1) from a methyl to an ethyl group (Table 4, entries 6–8). There was no reaction without the N-protection (Table 4, entry 1). Heating of the reaction mixture of 4-bromo-1-(phenylsulfonyl)-1*H*-pyrrolo[2,3-*b*]pyridine (**1b**) with phenylmethanamine (**4a**) in the presence of base and palladium catalyst resulted in the desulfonated 4-bromo-7-azaindole as the sole product (Table 4, entry 2).

**Table 4:** C–N-bond-formation cross coupling of N-protected 4-bromo-7-azaindoles **1** with amines **4**.

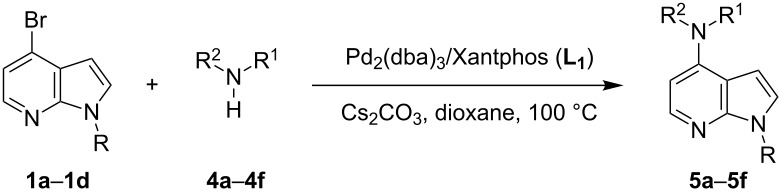					

Entry	7-Azaindole **1**	Amine **4**	Product **5**^a^	Time (h)	Yield (%)^b^

1	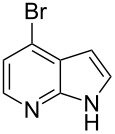 **1a**	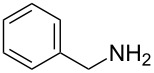 **4a**	–	24	NR^c^
2	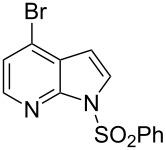 **1b**	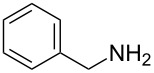 **4a**	–	3	0^d^
3	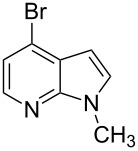 **1c**	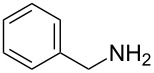 **4a**	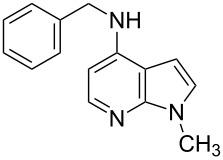 **5a**	2.5	92
4	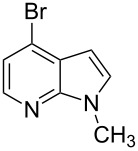 **1c**	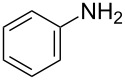 **4b**	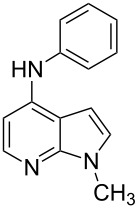 **5b**	3	91
5	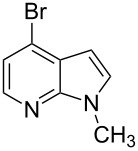 **1c**	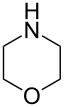 **4c**	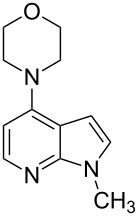 **5c**	3	88
6	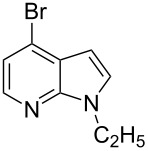 **1d**	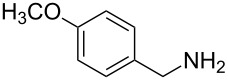 **4d**	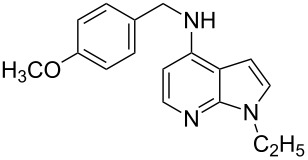 **5d**	2.5	93
7	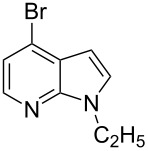 **1d**	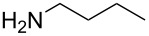 **4e**	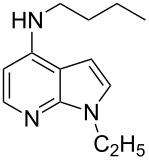 **5e**	2.5	90
8	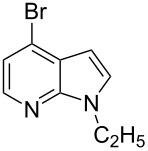 **1d**	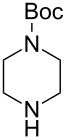 **4f**	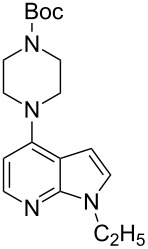 **5f**	3	94

^a^All reactions were carried out at 100 °C by using N-substituted 4-bromo-azaindoles **1** (1.0 mmol), amines (1.2 mmol), Cs_2_CO_3_ (1.5 mmol), Pd_2_(dba)_3_ (5 mol %), Xantphos (10 mol %), and 2 mL of dioxane. ^b^Yields reported are isolated yields. ^c^NR: no reaction. ^d^Desulfonation reaction takes place.

Our continuous efforts to develop synthetic methods for the formation of C–N bonds by coupling of N-protected 7-azaindoles **1** with amino acids or esters result in the development of interesting intermediates in our own medicinal chemistry program based on 7-azaindole. Large molecular architectures designed by cross-coupling strategies with the introduction of an amino acid functionality on 7-azaindole, result in new scaffolding. N-aryl-amino acids are reported as important synthetic intermediates and structural motifs for various drug-development programs by various medicinal and process-research chemists. Therefore, transition metal-catalyzed coupling of amino acids and its derivatives finds popularity in various coupling protocols [58]. A copper(I) iodide catalyzed coupling reaction of haloindoles with α-amino acids was reported by Ishikawa et al. [59].

Indole and azaindole moieties functionalized with amino acid ester scaffold are believed to be important synthetic intermediates and structural components of various medicinal and pharmaceutical candidates. The coupling of *N*-methyl-4-bromo-7-azaindole (**1c**) with D-alanine methyl ester **6b** was chosen as the model reaction to test the feasibility of the palladium-assisted coupling reaction of 7-azaindole and amino acids. The experimental results are summarized in Table 5. In our initial endeavor the coupling of *N*-methyl-4-bromo-7-azaindole (**1c**) with D-alanine (**6a**) resulted in only a trace amount of product **7a** with Xantphos (**L_1_**) as a ligand (Table 5, entry 1)**_._** Other bidentate aryl phosphine ligands **L_2_** and **L_3_** did not result in any product formation (Table 5, entries 2 and 3). The tertiary phosphine ligand PCy_3_ (**L_4_**) was ineffective in the arylation of *N*-methyl-4-bromo-7-azaindole (**1c**) with D-alanine (**6a**) (Table 5, entry 4). It is believed that the coordination of the central metal of the oxidative addition complex with the carboxyl functionality of the amino acid scaffold retained the Pd–N bond, making the 7-azaindole–Pd–N complexes too stable for reductive elimination [58]. As can be seen from Table 5, the reaction of *N*-methyl-4-bromo-7-azaindole (**1c**) with D-alanine methyl ester (**6b**) occurred rapidly with Pd_2_(dba)_3_ as a catalyst, Xantphos (**L_1_**) as ligand, and Cs_2_CO_3_ as base in dioxane at 100 °C in a short reaction time of 1 h (Table 5, entry 5). When K_2_CO_3_ was used as a base with Pd_2_(dba)_3_ as a catalyst, and Xantphos (**L_1_**) as a ligand, 85% of the product conversion was observed in 3 h (Table 5, entry 6). The other palladium catalyst Pd(OAc)_2_ results in poor yields of the product (Table 5, entry 13–19). Coupling of *N*-methyl-4-bromo-7-azaindole (**1c**) with D-alanine methyl ester (**6b**) by using SPhos (**L_2_**) as a ligand results in low product yield ~14% (Table 5, entry 10). When the bulkier ligand XPhos (**L_3_**) was used as a ligand, with Pd_2_(dba)_3_ as palladium source, and Cs_2_CO_3_ as base, a trace amount of product was formed after 6 h (Table 5, entry 11). On conducting the experiment with Pd(OAc)_2_ as catalyst and using the same ligand **L_3_**, no product formation was observed even after 24 h (Table 5, entry 18). The tertiary phosphine ligand PCy_3_ (**L_4_**) was found to be ineffective when treated with *N*-methyl-4-bromo-7-azaindole (**1c**) and D-alanine methyl ester (**6b**) (Table 5, entry 19). These results indicate that increasing the steric hindrance of the ligands promoted the reductive elimination step during the C–N-bond-forming step [58]. All the coupling reactions of amino acid esters were performed in dioxane as the solvent. Finally, Cs_2_CO_3_ as base (Table 5, entry 5) was found to be more effective than stronger bases such as NaO*t*-Bu, KOH and potassium phosphates (Table 5, entries 7–9).

**Table 5:** Optimization of the coupling reaction of **1c** with D-alanine methyl ester (**6b**).^a^

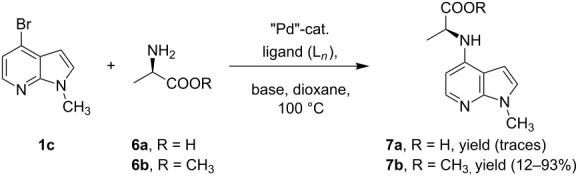						

Entry	Pd catalyst (5 mol %)	Amino acid (ester) **6**	L*_n_*	Base	Time (h)	Yield (%)^b^

1	Pd_2_(dba)_3_	**6a**	**L_1_**	Cs_2_CO_3_	24	traces^c^
2	Pd_2_(dba)_3_	**6a**	**L_2_**	Cs_2_CO_3_	24	0
3	Pd_2_(dba)_3_	**6a**	**L_3_**	Cs_2_CO_3_	24	0
4	Pd_2_(dba)_3_	**6a**	**L_4_**	Cs_2_CO_3_	24	0
5	Pd_2_(dba)_3_	**6b**	**L_1_**	Cs_2_CO_3_	1	93
6	Pd_2_(dba)_3_	**6b**	**L_1_**	K_2_CO_3_	3	85
7	Pd_2_(dba)_3_	**6b**	**L_1_**	NaO*t*-Bu	3	44
8	Pd_2_(dba)_3_	**6b**	**L_1_**	KOH	3	33
9	Pd_2_(dba)_3_	**6b**	**L_1_**	K_3_PO_4_	3	12
10	Pd_2_(dba)_3_	**6b**	**L_2_**	Cs_2_CO_3_	6	14
11	Pd_2_(dba)_3_	**6b**	**L_3_**	Cs_2_CO_3_	6	traces
12	Pd_2_(dba)_3_	**6b**	**L_4_**	Cs_2_CO_3_	24	0
13	Pd(OAc)_2_	**6b**	**L_1_**	Cs_2_CO_3_	24	20
14	Pd(OAc)_2_	**6b**	**L_1_**	K_2_CO_3_	24	15
15	Pd(OAc)_2_	**6b**	**L_1_**	NaO*t*-Bu	24	23
16	Pd(OAc)_2_	**6b**	**L_1_**	K_3_PO_4_	24	20
17	Pd(OAc)_2_	**6b**	**L_2_**	Cs_2_CO_3_	24	18
18	Pd(OAc)_2_	**6b**	**L_3_**	Cs_2_CO_3_	24	0
19	Pd(OAc)_2_	**6b**	**L_4_**	Cs_2_CO_3_	24	0

^a^Reaction conditions: *N*-methyl-4-bromo-7-azaindole (**1c**) (1.0 mmol), amino acid (ester) (1.2 mmol), base (3.0 mmol), palladium catalyst (5 mol %), ligand (10 mol %), and 2 mL of dioxane, 100 °C, 1–24 h. ^b^Yields reported are isolated yields. ^c^Trace amount of product obtained by cross coupling of **1c** with **6a**.

With a viable coupling procedure in hand, attention was turned to the generality of the process and couplings of structurally diverse amino acid building blocks. Results summarized in Table 6 show that the optimized conditions described proved to be general for the coupling with a wide variety of amino acid building blocks. As can be seen from Table 6, the catalytic system works well with diversified amino acid building blocks. Coupling of *N*-methyl-4-bromo-7-azaindole (**1c**) with D-alanine methyl ester (**6b**) resulted in good yield of the product **7b** in a short reaction time (Table 6, entry 2). The chiral purity of the product was determined by chiral HPLC using Chiral Pak AD-H column. Amino acids without extra coordinating groups gave good coupling yields (Table 6, entries 3, 6 and 7). Coupling of L-serine(*O*-*t*-Bu)-OMe (**6d**) with **1c** resulted in moderate yield of the product in 3 h (Table 6, entry 4). The catalytic system developed by us for the coupling of amino acid esters with N-protected 7-azaindoles was ineffective for L-proline (**6f**), L-serine (**6g**), and L-glutamic acid (**6h**) (Table 6, entries 8–10). This may be ascribed to the fact that these amino acids contain more heteroatoms that bind to the central palladium atom and enhance the stability of the 7-azaindole–Pd–N complexes, making them too stable for reductive elimination.

**Table 6:** C–N-bond-formation cross coupling of N-protected 4-bromo-7-azaindoles **1** with amino acid (esters) **6**.

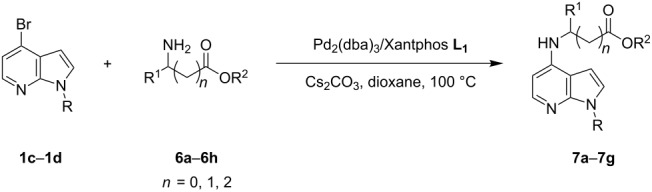						

Entry	7-Azaindole **1**	Amino acid (ester) **6**	Product **7**^a^	Time (h)	Yield (%)^b^	ee (%)^d^

1	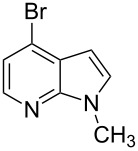 **1c**	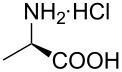 **6a**	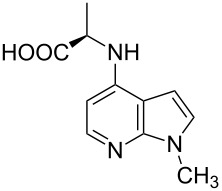 **7a**	2	traces	–
2	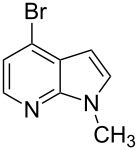 **1c**	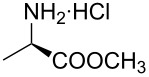 **6b**	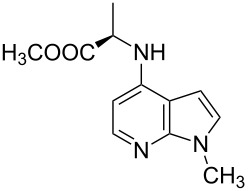 **7b**	2	70	98.79
3	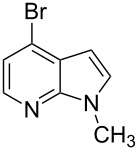 **1c**	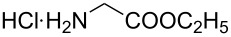 **6c**	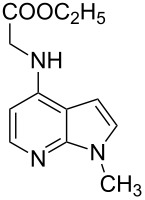 **7c**	2	72	–
4	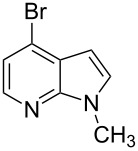 **1c**	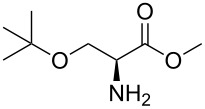 **6d**	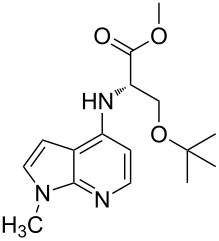 **7d**	3	65	95.48
5	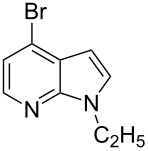 **1d**	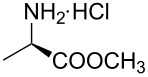 **6b**	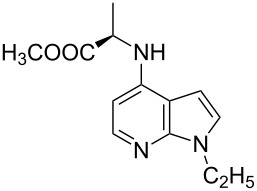 **7e**	2	71	98.91
6	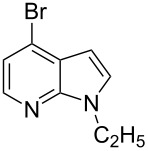 **1d**	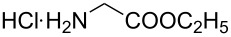 **6c**	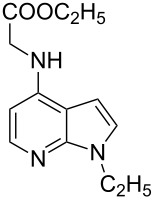 **7f**	2.1	72	–
7	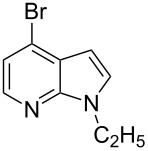 **1d**	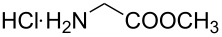 **6e**	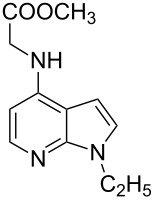 **7g**	2	70	–
8	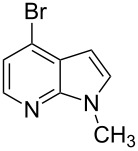 **1c**	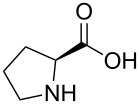 **6f**	–	5	0	–
9	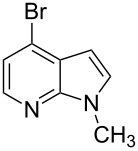 **1c**	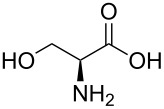 **6g**	–	5	0	–
10	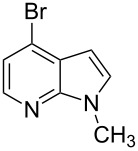 **1c**	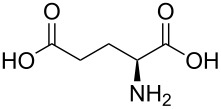 **6h**	–	5	0	–

^a^All reactions were carried out at 100 °C. N-substituted 4-bromo-azaindoles **1c** or **1d** (1.0 mmol), amino acid (esters) (1.2 mmol), Cs_2_CO_3_ (3.0 mmol), Pd_2_(dba)_3_ (5 mol %) and Xantphos (10 mol %) were used for all the reactions. ^b^Yields reported are isolated yields. ^c^Desulfonation reaction takes place. ^d^ee was determined by chiral HPLC.

After successful demonstration of the C–N-bond-formation reaction of 4-bromo-7-azaindole derivatives with amides, amines and amino acid esters, we wanted to expand the scope of the reaction towards C–O-bond formation. Until today no general method has been described for the C–O-bond-formation reaction of 4-halo-azaindole with phenols or alcohols. Most of the literature reports described on C–O-bond-formation reactions are limited to aryl halides and phenols or alcohols only [36,60–63]. Functionalization of 4-substituted-7-azaindole scaffolds with 4-amino-2-fluorophenol was reported to be ineffective upon heating in the presence of a strong base such as KO*t*-Bu [35]. In addition, the *N*-oxide derivative of 4-substituted 7-azaindole fails to provide the desired product under similar conditions [48]. Further, on utilizing palladium or copper-mediated cross-coupling reactions of N-protected amino-2-fluorophenol with 4-chloro- or 4-bromo-1*H*-pyrrolo[2,3-*b*]pyridine, the desired diaryl ether could not be isolated in acceptable yield [5,35]. To select the best reaction conditions for C–O-bond formation, we envisaged the synthesis of an activated 7-azaindole building block that could be coupled with phenols. To select the best coupling conditions for C–O bond formation, the coupling of 4-bromo-1-methy-1*H*-pyrrolo[2,3-*b*]pyridine (**1c**) with *m*-cresol (**8a**) was selected as a model reaction to find the suitable ligands (Scheme 1), palladium catalysts, bases and organic solvents. The experimental findings are summarized in Table 7. The coupling of **1c** with **8a** by using a combination of Pd(OAc)_2_, Xantphos (**L_1_**) and K_2_CO_3_ in dioxane at 100 °C in 10 h of time provided 70% of the desired diaryl ether **9a** (Table 7, entry 3). The reaction rate is slow, i.e., when run for 3 h at 100 °C, only 30% product was obtained (Table 7, entry 2). But upon continuous heating for 7 h we observed 70% (Table 7, entry 3) of the expected product. Interestingly, usage of Pd_2_(dba)_3_ resulted in poor yields of the product (Table 7, entries 5 and 6). In most of the cases we observed decomposition of the Pd_2_(dba)_3_ reagent. In comparison to the conditions described for the amines and amides, a much longer reaction time was required for the C–O-bond formation when treated with phenols. K_2_CO_3_ was found to be a suitable base for the C–O-bond formation under the experimental conditions we studied. When Cs_2_CO_3_ was used as base, settling of the base was observed even under heating and stirring of the reaction mixture at 100 °C. The probable reason may be that Cs_2_CO_3_ is much heavier than K_2_CO_3_ and tends to settle in the bottom of the reaction vessel or reactor when run on a larger scale, causing improper mixing of the heterogeneous mixture.

**Table 7:** Optimization of the coupling reaction of *N*-methyl-4-bromo-7-azaindole (**1c**) with *m*-cresol (**8a**).^a^

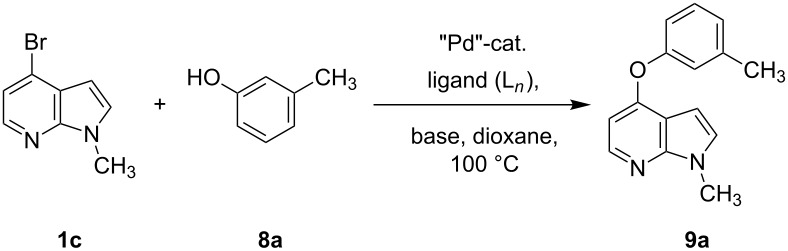						

Entry	Pd catalyst (5 mol %)	L*_n_*	Base	Solvent	Time (h)	Yield (%)^b^

1	Pd(OAc)_2_	**L_1_**	Cs_2_CO_3_	dioxane	5	20
2	Pd(OAc)_2_	**L_1_**	K_2_CO_3_	dioxane	3	30
3	Pd(OAc)_2_	**L_1_**	K_2_CO_3_	dioxane	10	70
4	Pd(OAc)_2_	**L_1_**	K_2_CO_3_	THF	10	50
5	Pd_2_(dba)_3_	**L_1_**	Cs_2_CO_3_	dioxane	3	10
6	Pd_2_(dba)_3_	**L_1_**	K_2_CO_3_	dioxane	10	32
7	Pd(OAc)_2_	**L_2_**	Cs_2_CO_3_	dioxane	24	45
7	Pd(OAc)_2_	**L_3_**	Cs_2_CO_3_	dioxane	24	61
9	Pd(OAc)_2_	**L_1_**	K_2_CO_3_	dioxane	12	68
10	Pd(OAc)_2_	**L_1_**	K_2_CO_3_	dioxane	24	65

^a^Reaction conditions: *N*-methyl-4-bromo-7-azaindole (**1c**) (1.0 mmol), *m*-cresol (1.2 mmol), base (3.0 mmol), palladium catalyst (5 mol %), ligand (10 mol %), and 2 mL of dioxane, 100 °C, 3–24 h. ^b^Yields reported are isolated yields.

With a viable coupling procedure in hand, attention was turned to the generality of the process and couplings of structurally diverse phenols. Results are summarized in Table 8. The C–O-bond formation was established with good yields with phenol derivatives and 1-naphthol (Table 8, entries 1–3). Moreover, the outcome of the reaction strongly depended on the electronic character of the appropriate phenol (Table 8). The more-electron-rich nucleophiles **8a**, **8b** furnished the desired ethers **9a** and **9b** in good yields. Further studies are in progress in our laboratory to investigate different substrate scope and mechanistic aspects of the C–O-bond-forming reaction.

**Table 8:** C–O-bond-formation cross coupling of *N*-methyl-4-bromo-7-azaindole (**1c**) with phenols **9a**–**9c**.

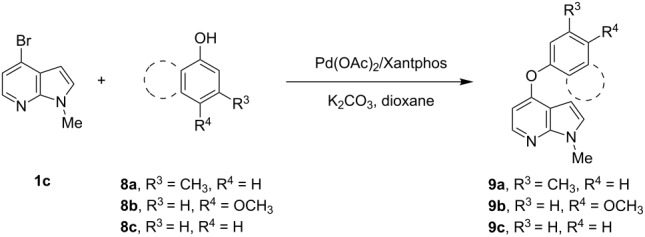					

Entry	7-Azaindole **1**	Phenol **8**	Product **9**^a^	Time (h)	Yield (%)^b^

1	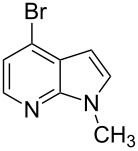 **1c**	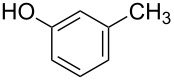 **8a**	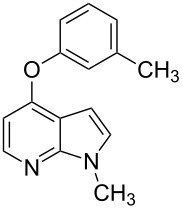 **9a**	10	70
2	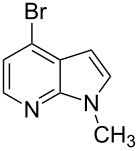 **1c**	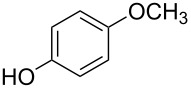 **8b**	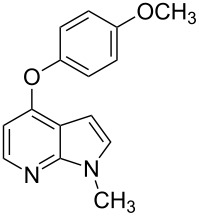 **9b**	8	85
3	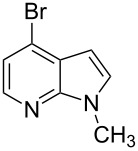 **1c**	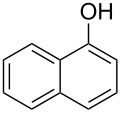 **8c**	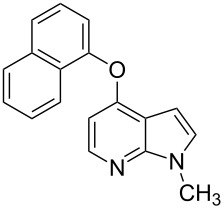 **9c**	11	72

^a^All reactions were carried out at 100 °C in a dried sealed Schlenk tube by using *N*-methyl-4-bromo-7-azaindole (**1c**) (1.0 mmol), phenol (1.2 mmol), K_2_CO_3_ (1.5 mmol), Pd(OAc)_2_ (5 mol %), Xantphos (10 mol %) and 2 mL of dioxane. ^b^Yields reported are isolated yields.

## Conclusion

In conclusion, we have developed the best coupling conditions for C–N-bond formation of N-substituted 4-bromo-7-azaindoles with amides, amines, and amino acid esters and demonstrated well for the synthesis of various N-substituted 7-azaindole compounds, which are very difficult to synthesize otherwise. The combination of Xantphos, Cs_2_CO_3_ and dioxane was found to be crucial for all the C–N cross-coupling reactions. However, different Pd-catalyst precursors were used for different amines/amides and amino acid esters. We have enhanced the methodology towards the C–O-bond formation with various phenols, which is very difficult to achieve. K_2_CO_3_ was found to be better for C–O cross-coupling reactions. This protocol provides a nice alternative for the synthesis of N-substituted 7-azaindole derivatives, which exist extensively in natural products and pharmaceuticals. This is the first report on coupling of amides, amino acid esters and phenols with N-substituted 4-bromo-7-azaindole. Hence, we feel that our methodology will serve as an excellent tool in medicinal chemistry, organic synthesis and process research worldwide.

## Supporting Information

Supporting information, containing all experimental details and analytical data of all new compounds given in this article as well as their ^1^H, ^13^C NMR spectra and HRMS data, is provided.

File 1 Experimental procedures, analytical data and NMR spectra.

## References

[1] Zhao S-B, Wang S (2010). Chem Soc Rev.

[2] Popowycz F, Routier S, Joseph B, Mérour J-Y (2007). Tetrahedron.

[3] Song J J, Reeves J T, Gallou F, Tan Z, Yee N K, Senanayake C H (2007). Chem Soc Rev.

[4] Schlummer B, Scholz U (2004). Adv Synth Catal.

[5] Caldwell J J, Cheung K-W, Collins I (2007). Tetrahedron Lett.

[6] Schneller S W, Luo J-K (1980). J Org Chem.

[7] Girgis N S, Larson S B, Robins R K, Cottam H B (1989). J Heterocycl Chem.

[8] Guillard J, Decrop M, Gallay N, Espanel C, Boissier E, Herault O, Viaud-Massuard M-C (2007). Bioorg Med Chem Lett.

[9] Busacca C A, Fandrick D R, Song J J, Senanayake C H (2011). Adv Synth Catal.

[10] Merour J-Y, Joseph B (2001). Curr Org Chem.

[11] Buckingham J (1994). Dictionary of Natural Products.

[12] Czarnik A W (1996). Acc Chem Res.

[13] Theil F (1999). Angew Chem, Int Ed.

[14] Sawyer J S (2000). Tetrahedron.

[15] Ley S V, Thomas A W (2003). Angew Chem, Int Ed.

[16] Cristau P, Vors J-P, Zhu J (2003). Tetrahedron.

[17] Muci A R, Buchwald S L (2002). Top Curr Chem.

[18] Yin J, Buchwald S L (2000). Org Lett.

[19] Fors B P, Krattiger P, Strieter E, Buchwald S L (2008). Org Lett.

[20] Wolfe J P, Tomori H, Sadighi J P, Yin J, Buchwald S L (2000). J Org Chem.

[21] Sadig J E R, Willis M C (2011). Synthesis.

[22] Hooper M W, Utsunomiya M, Hartwig J F (2003). J Org Chem.

[23] Peng Z-H, Journet M, Humphrey G (2006). Org Lett.

[24] Chen W, Li J, Fang D, Feng C, Zhang C (2008). Org Lett.

[25] Zhang H, Cai Q, Ma D (2005). J Org Chem.

[26] Fors B P, Dooleweerdt K, Zeng Q, Buchwald S L (2009). Tetrahedron.

[27] Hartwig J F (2008). Acc Chem Res.

[28] Buchwald S L, Mauger C, Mignani G, Scholz U (2006). Adv Synth Catal.

[29] Messaoudi S, Audisio D, Brion J-D, Alami M (2007). Tetrahedron.

[30] Vimolratana M, Simard J L, Brown S P (2011). Tetrahedron Lett.

[31] Yao P-Y, Zhang Y, Hsung R P, Zhao K (2008). Org Lett.

[32] Schwarz N, Pews-Davtyan A, Alex K, Tillack A, Beller M (2007). Synthesis.

[33] Benarous R, Barbey-Treve S, Paris J-M, Berrut S, Berlioz-Torrent C, Emiliani S (2010). Novel substituted aryl derivatives, their process of preparation and their therapeutical uses as anti-HIV agents. WO Patent.

[34] Sergeev A G, Artamkina G A, Velezheva V S, Fedorova I N, Beletskava I P (2005). Russ J Org Chem.

[35] Edmondson S D, Mastracchio A, Parmee E R (2000). Org Lett.

[36] Gowrisankar S, Sergeev A G, Anbarasan P, Spannenberg A, Neumann H, Beller M (2010). J Am Chem Soc.

[37] Thutewohl M, Schirok H, Bennabi S, Figueroa-Pérez S (2006). Synthesis.

[38] Figueroa-Pérez S, Bennabi S, Schirok H, Thutewohi M (2006). Tetrahedron Lett.

[39] Henderson J L, McDermott S M, Buchwald S L (2010). Org Lett.

[40] Blake J, Gunawardana I W, Le Huerou Y, Mohr P J, Wallace E M, Wang B (2009). Pyrrolopyridines as kinase inhibitors. WO Patent.

[41] Toshihiro H, Hiroko N, Jun T (2010). Novel compounds. U.S. Patent Application.

[42] Surasani R, Kalita D, Rao A V D, Yarbagi Y, Chandrasekhar K B (2012). J Fluorine Chem.

[43] Layek M, Gajare V, Kalita D, Islam A, Mukkanti K, Pal M (2009). Tetrahedron.

[44] Layek M, Gajare V, Kalita D, Islam A, Mukkanti K, Pal M (2009). Tetrahedron Lett.

[45] Layek M, Rao A V D, Gajare V, Kalita D, Barange D K, Islam A, Mukkanti K, Pal M (2009). Tetrahedron Lett.

[46] Layek M, Lakshmi U, Kalita D, Barange D K, Islam A, Mukkanti K, Pal M (2009). Beilstein J Org Chem.

[47] Thibault C, L'Heureux A, Bhide R S, Ruel R (2003). Org Lett.

[48] Kühler T C, Swanson M, Shcherbuchin V, Larsson H, Mellgård B, Sjöström J-E (1998). J Med Chem.

[49] Antilla J C, Klapars A, Buchwald S L (2002). J Am Chem Soc.

[50] Old D W, Harris M C, Buchwald S L (2000). Org Lett.

[51] Kranenburg M, van der Burgt Y E M, Kamer P C J, van Leeuwen P W N M, Goubitz K, Fraanje J (1995). Organometallics.

[52] Guari Y, van Es D S, Reek J N H, Kamer P C J, van Leeuwen P W N M (1999). Tetrahedron Lett.

[53] Wagaw S, Yang B H, Buchwald S L (1999). J Am Chem Soc.

[54] Imbriglio J E, DiRocco D, Raghavan S, Ball R G, Tsou N, Mosley R T, Tata J R, Colletti S L (2008). Tetrahedron Lett.

[55] Vandromme L, Legraverend M, Kreimerman S, Lozach O, Meijer L, Grierson D S (2007). Bioorg Med Chem.

[56] Piguel S, Legraverend M (2007). J Org Chem.

[57] Chaulet C, Croix C, Basset J, Pujol M-D, Viaud-Massuard M-C (2010). Synlett.

[58] Ma F, Xie X, Ding L, Gao J, Zhang Z (2011). Tetrahedron.

[59] Kurokawa M, Nakanishi W, Ishikawa T (2007). Heterocycles.

[60] Wolter M, Nordmann G, Job G E, Buchwald S L (2002). Org Lett.

[61] Kuwabe S-i, Torraca K E, Buchwald S L (2001). J Am Chem Soc.

[62] Torraca K E, Huang X, Parrish C A, Buchwald S L (2001). J Am Chem Soc.

[63] Parrish C A, Buchwald S L (2001). J Org Chem.

