# Supplementary material for: Palladium-catalyzed C–N and C–O bond formation of N-substituted 4-bromo-7-azaindoles with amides, amines, amino acid esters and phenols
Source: Beilstein J Org Chem. 2012 Nov 19;8:2004–18. doi: 10.3762/bjoc.8.227 (PMC3511036; doi:10.3762/bjoc.8.227)

Supporting Information

for

Palladium-catalyzed C–N and C–O bond formation of N-substituted 4-bromo-7-azaindoles with amides, amines, amino acid esters and phenols

Rajendra Surasani[1,2], Dipak Kalita*[1], A. V. Dhanunjaya Rao[1] and K. B. Chandrasekhar[2]

Address: [1]Custom Pharmaceutical Services, Dr. Reddy's Laboratories Ltd., Miyapur, Hyderabad – 50049, India and [2]Department of Chemistry, Institute of Science and Technology, JNTU University of Anantapur, Anantapur 515002, India

Email: Dipak Kalita - dipakk@drreddys.com

*Corresponding author

[#]DRL Communication no.: IPDO IPM- 00294

Experimental procedures, analytical data and NMR spectra

Table of Contents .... Page

General Information S2
Experimental procedures and analytical data S2–S11
^{1}H and ^{13}C NMR spectra S12–S32

General Information: All solvents were distilled prior to use. $Pd_2(dba)_3$, $Pd(OAc)_2$, Xantphos, XPhos, SPhos, amines, amino acids and amides were purchased from Aldrich and Alfa Aesar. Anhydrous solvents were distilled by following standard protocols. Melting points (mp) were recorded with a Büchi melting point B-540 instrument and are uncorrected. IR spectra were recorded as KBr pellet with a Shimadzu IR-Prestige-21 instrument and only diagnostic and/or intense peaks are reported. Mass spectra were recorded with a PE Sciex model API 3000 instrument. HRMS spectra were recorded with Waters LCT Premier XE (Micro mass Oa-TOF) instrument. ^{1}H NMR spectra were recorded in DMSO-d_6 with a Varian Mercury plus 400 and 500 MHz instrument. ^{13}C NMR spectra were recorded in DMSO-d_6 with a Varian Gemini 200 MHz instrument. Signals due to the solvent (^{13}C NMR) or residual protonated solvent (^{1}H NMR) served as the internal standard. The ^{1}H NMR chemical shifts and coupling constants were determined assuming first-order behavior. Multiplicity is indicated by one or more of the following: s (singlet), d (doublet), t (triplet), q (quartet), m (multiplet), br (broad); the list of coupling constants (*J*) corresponds to the order of multiplicity assignment.

General procedure for C–N bond formation by coupling of 4-bromo-7-azaindole derivatives with amides

To a 100 mL dried sealed Schlenk tube charged N-substituted 4-bromo 7-azaindole (1.0 mmol), amide (1.2 mmol), cesium carbonate (1.5 mmol), $Pd(OAc)_2$ (5 mol %), Xantphos (10 mol %), and 2 mL of dioxane were added. Nitrogen was bubbled through the reaction mass for 2 min. The reaction mixture was heated to 100 °C and stirred for the appropriate time as mentioned in Table 2. The reaction mass was

cooled to room temperature and diluted with ethyl acetate (20 mL), filtered through a celite bed and wash with ethyl acetate (10 mL). The filtrate was concentrated in vacuum. The crude product was purified by column chromatography on silica gel (100–200) using ethyl acetate and hexane mixture as an eluent to afford the pure title products.

***N*-(1-methyl-1*H*-pyrrolo[2,3-*b*]pyridin-4-yl)benzamide (3a).** Off white solid; mp 145–147 °C; IR (KBr): 3400, 3178, 3061, 1678, 1656, 1608, 1575, 1498, 1394, 1317, 713, 555 cm^{-1}; ^{1}H NMR (400 MHz, DMSO-d_6): δ 10.42 (brs, 1H. NH), 8.20 (d, *J* = 5.2 Hz, 1H), 7.99-7.97 (m, 2H), 7.72 (d, *J* = 5.2 Hz, 1H), 7.64-7.53 (m, 4H), 7.41 (d, *J* = 3.6 Hz, 1H), 6.81 (d, *J* = 3.6 Hz, 1H), 3.81 (s, 3H, N-CH_3); ^{13}C NMR (50 MHz, DMSO-d_6): δ 166.41, 148.75, 142.96, 138.29, 134.61, 131.73, 128.28, 128.05, 127.90, 111.76, 106.87, 98.08, 30.95; MS (ES): m/z = 262.4 (M+1); HRMS (ESI): calculated for $C_{15}H_{14}N_3O_2$ $(M+H)^+$ 262.1126; found 262.1137.

***N*-(1-methyl-1*H*-pyrrolo[2,3-*b*]pyridin-4-yl)benzene sulfonamide (3b).** Off white solid; mp 175-177 °C; IR (KBr): 3259, 3111, 1604, 1571, 1517, 1444, 1398, 1330, 1309, 1161, 1091, 894, 713, 580 cm^{-1}; ^{1}H NMR (400 MHz, DMSO-d_6): δ 10.88 (brs, 1H. NH), 8.03 (d, *J* = 5.6 Hz, 1H), 7.90 (d, *J* = 2.0 Hz, 1H), 7.87 (d, *J* = 1.6 Hz, 1H), 7.52-7.60 (m, 3H), 7.33 (d, *J* = 3.6 Hz, 1H), 6.99 (d, *J* = 5.6 Hz, 1H), 6.78 (d, *J* = 3.6 Hz, 1H), 3.72 (s, 3H, N-CH_3); ^{13}C NMR (50 MHz, DMSO-d_6): δ 143.02, 139.61, 133.08, 128.28, 128.83, 128.18, 126.56, 125.50, 110.77, 103.50, 97.17, 30.96; MS (ES): m/z = 288.3 (M+1); HRMS (ESI): calculated for $C_{14}H_{14}N_3O_2S$ $(M+H)^+$ 288.0807; found 288.0820.

1-(1-methyl-1*H*-pyrrolo[2,3-*b*]pyridin-4-yl)pyrrolidin-2-one (3c). Brown thick liquid; IR (KBr): 2924, 1705, 1568, 1384, 1309, 823, 754, 721 cm^{-1}; ^{1}H NMR (400 MHz, DMSO-d_6): δ 8.19 (d, *J* = 5.2 Hz, 1H), 7.45 (d, *J* = 3.6 Hz, 1H), 7.34 (d, *J* = 5.2 Hz,

1H), 6.52 (d, *J* = 3.6 Hz, 1H), 4.05 (t, *J* = 6.8 Hz, 2H), 3.80 (s, 3H, N-CH_3), 2.50 (t, *J* = 2.0 Hz, 2H), 2.16-2.09 (m, 2H); ^{13}C NMR (50 MHz, DMSO-d_6): δ 173.98, 149.10, 142.55, 139.13, 128.42, 112.45, 107.72, 99.32, 49.40, 31.78, 31.00, 18.58.; MS (ES): m/z = 216.3 (M+1).

***N*-(1-ethyl-1*H*-pyrrolo[2,3-*b*]pyridin-4-yl)-2-methoxybenzamide (3d).** Off white solid; ^{1}H NMR (400 MHz, DMSO-d_6): δ 10.44 (brs, 1H. NH), 8.20 (d, *J* = 5.2 Hz, 1H), 8.08 (d, *J* = 4.8 Hz, 1H), 7.82 (d, *J* = 3.6 Hz, 1H), 7.65 (d, *J* = 4.2 Hz, 1H), 7.53 (m,1H), 7.22 (d, *J* = 3.4 Hz, 1H), 7.18 (t, *J* = 7.2 Hz, 1H), 6.68 (d, *J* = 3.6 Hz, 1H), 4.30 (m, 2H), 4.03 (s, 3H), 139 (t, 3H, *J* = 7.2 Hz); ^{13}C NMR (50 MHz, DMSO-d_6): δ 164.22, 157.11, 147.82, 143.34, 137.77, 132.98, 130.60, 126.73, 122.78, 120.78, 112.30, 110.52, 105.14, 96.02, 56.26, 55.69, 15.43; MS (ES): m/z = 296.30 (M+1); HRMS (ESI): calculated for $C_{17}H_{18}N_3O_2$ $(M+H)^+$ 296.1399; found 296.1397.

***N*-(1-ethyl-1*H*-pyrrolo[2,3-*b*]pyridin-4-yl)-4-fluorobenzamide (3e).** Off white solid; ^{1}H NMR (500 MHz, DMSO-d_6): δ 10.47 (brs, 1H. NH), 8.20 (d, *J* = 4.4 Hz, 1H), 8.08 (d, *J* = 4.8 Hz, 1H), 8.06 (d, *J* = 4.4Hz, 1H), 7.69 (d, *J* = 4.4 Hz, 1H), 7.49 (d, *J* = 2.8 Hz, 1H), 7.41 (d, *J* = 7.2 Hz, 2H), 6.81 (d, *J* = 2.8 Hz, 1H), 4.31 (m, 2H), 1.39 (t, 3H, *J* = 6.0 Hz); ^{13}C NMR (100 MHz, DMSO-d_6): δ 165.38, 148.24, 142.92, 138.22, 131.14, 131.11, 130.97, 130.88, 126.54, 115.41, 115.19, 112.04, 107.08, 98.28, 38.80, 15.45; MS (ES): m/z = 284.30 (M+1); HRMS (ESI): calculated for $C_{16}H_{15}N_3OF$ $(M+H)^+$ 284.1199; found 284.1198.

2-Amino-*N*-(1-ethyl-1*H*-pyrrolo[2,3-*b*]pyridin-4-yl)benzamide (3f). Light yellow solid; ^{1}H NMR (400 MHz, DMSO-d_6): δ 10.51 (s, 1H), 8.16-8.15 (d, *J* = 5.2 Hz, 1H), 7.69-7.67 (d, *J* = 7.6 Hz, 1H), 7.62-7.60 (d, *J* = 5.2 Hz, 1H), 7.46-7.45 (d, *J* = 3.6 Hz, 1H), 7.26-7.22 (t, *J* = 6.8 Hz, 1H), 6.79-6.75 (m, 2H), 6.64-6.60 (t, *J* = 7.6 Hz, 1H), 6.33 (s, 2H), 4.30-4.25 (m, 2H), 1.39-1.35 (t, *J* = 7.6 Hz, 3H); ^{13}C NMR (100 MHz,

DMSO-d_6): δ 168.36, 149.90, 148.21, 142.88, 138.68, 132.57, 129.53, 126.44, 116.48, 115.07, 114.92, 112.13, 107.08, 98.39, 38.87, 15.62; MS (ES): m/z = 281.20 (M+1); HRMS (ESI): calculated for $C_{16}H_{17}N_4O$ $(M+H)^+$ 281.1402; found 281.1395.

***N*-(1-benzyl-1*H*-pyrrolo[2,3-*b*]pyridin-4-yl)benzamide (3g).** Light greenish solid; mp 165-167 °C; IR (KBr): 3244, 2924, 1876, 1654, 1575, 1346, 1307, 1055, 902, 821, 721, 704, 557 cm^{-1}; ^1H NMR (400 MHz, DMSO-d_6): δ 10.44 (brs, s, 1H, NH), 8.20 (d, *J* = 5.2 Hz, 1H), 7.98-7.96 (m, 2H), 7.74 (d, *J* = 5.2 Hz, 1H), 7.65-7.54 (m, 3H), 7.53 (d, *J* = 3.6 Hz, 1H), 7.36-7.21 (m, 4H), 6.87 (d, *J* = 3.2 Hz, 1H), 5.48 (s, 2H, -N-CH2-); ^{13}C NMR (50 MHz, DMSO-d_6): δ 166.46, 148.50, 143.24, 138.51, 138.45, 134.60, 131.79, 128.40, 128.32, 128.06, 127.21, 127.13, 111.86, 107.23, 98.90, 47.23; MS (ES): m/z =328.4 (M+1); HRMS (ESI): calculated for $C_{21}H_{18}N_3O$ $(M+H)^+$ 328.1450; found328.1459.

General procedure for C-N bond formation by coupling of 4-bromo-7-azaindole derivatives with amines

To a 100 mL dried sealed Schlenk tube, N-substituted 4-bromo azaindole (1.0 mmol), amine (1.2 mmol), cesium carbonate (1.5 mmol), $Pd_2(dba)_3$ (5 mole %), Xantphos (10 mole %), and 2 mL of dioxane were added. Nitrogen gas was bubbled through the reaction mass for 10 minutes. The reaction mixture was heated to 100 °C and stirred for appropriate time as mentioned in Table no. 4. The reaction mass was cooled to room temperature and diluted with ethyl acetate (20 mL), filtered through a celite bed and wash with ethyl acetate (10 mL).The filtrate was concentrated in vacuum. The crude product was purified by column chromatography over silica gel (100-200) using ethyl acetate and hexane mixture as an eluent to afford the pure title products.

***N*-benzyl-1-methyl-1*H*-pyrrolo[2,3-*b*]pyridin-4-amine (5a).** Light-brown solid; mp 136-138 °C; IR (KBr): 3238, 3028, 1604, 1504, 1336, 1305, 1103, 1076, 869, 707, 623 cm^{-1}; ^{1}H NMR (400 MHz, DMSO-d_6): δ 7.77 (d, *J* = 5.6 Hz, 1H), 7.36-7.28 (m, 4H), 7.23-7.18 (m, 2H), 7.11 (d, *J* = 3.2 Hz, 1H), 6.60 (d, *J* = 3.6 Hz, 1H), 6.07 (d, *J* = 5.6 Hz, 1H), 4.48 (d, *J* = 6.0 Hz, 2H), 3.69 (s, 3H, N-CH3); ^{13}C NMR (50 MHz, DMSO-d_6): δ 147.92, 147.46, 143.86, 139.74, 128.23, 126.81, 126.60, 124.70, 107.44, 96.89, 96.18, 45.48, 30.80; MS (ES): m/z = 238.4 (M+1); HRMS (ESI): calculated for $C_{15}H_{16}N_3$ $(M+H)^+$ 238.1344; found 238.1348.

***N*-phenyl-1-methyl-1*H*-pyrrolo[2,3-*b*]pyridin-4-amine (5b).** Light-brown solid; mp 220.-224 °C; IR (KBr): 3238, 3095, 1610, 1570, 1490, 1330, 1240, 1207, 729, 646 cm^{-1}; ^{1}H NMR (400 MHz, DMSO-d_6): δ 8.62 (brs, 1H. NH), 7.94 (d, *J* = 5.6 Hz, 1H), 7.37-7.27 (m, 5H), 7.24 (d, *J* = 3.2 Hz, 1H), 7.01-7.05 (m, 1H), 6.70 (d, *J* = 5.6 Hz, 1H), 6.60 (d, *J* = 3.6 Hz, 1H), 3.75 (s, 3H, N-CH3); ^{13}C NMR (50 MHz, DMSO-d_6): δ 148.72, 143.67, 141.07, 129.06, 125.97, 122.30, 120.62, 108.95, 98.62, 97.27, 30.93; MS (ES): m/z = 224.2 (M+1); HRMS (ESI): calculated for $C_{14}H_{14}N_3$ $(M+H)^+$ 224.1188; found 224.1186.

4-(1-methyl-1*H*-pyrrolo[2,3-*b*]pyridin-4-yl)morpholine (5c). Brown solid; mp 82-84 °C; IR (KBr): 2954, 2816, 1874, 1575, 1355, 1309, 1251, 1112, 991, 812, 709, 628 cm^{-1}; ^{1}H NMR (400 MHz, DMSO-d_6): δ 8.01 (d, *J* = 5.2 Hz, 1H), 7.30 (d, *J* = 3.6 Hz, 1H), 6.50 (d, *J* = 3.6 Hz, 1H), 6.45 (d, *J* = 5.2 Hz, 1H), 3.79-3.77 (t, *J* = 4.8 Hz, 4H), 3.75 (s, 3H, N-CH_3), 3.37-3.35 (t, *J* = 4.8 Hz, 4H); ^{13}C NMR (50 MHz, DMSO-d_6): δ 150.78, 148.82, 143.49, 126.25, 109.78, 101.16, 98.57, 66.02, 49.07, 30.98; MS (ES): m/z = 218.3 (M+1); HRMS (ESI): calculated for $C_{12}H_{16}N_3O$ $(M+H)^+$ 218.1293; found 218.1291.

1-Ethyl-*N*-(4-methoxybenzyl)-1*H*-pyrrolo[2,3-*b*]pyridin-4-amine (5d). Light brown solid; mp 98-100 °C; IR (KBr): 3240, 2927, 1861, 1606, 1572, 1502, 1344, 1317, 1209, 1082, 935, 867, 794, 773, 723, 623 cm^{-1}; ^{1}H NMR (400 MHz, DMSO-d_6): δ 7.76 (d, J = 5.6 Hz, 1H), 7.29-7.26 (m, 2H), 7.16-7.12 (m, 2H), 6.89-6.85 (m, 2H), 6.58 (d, J = 3.2 Hz, 1H), 6.07 (d, J = 5.6 Hz, 1H), 4.39 (d, J = 6.0 Hz, 1H), 4.17-4.12 (q, J = 7.2 Hz, 2H, N-CH_2), 3.70 (s, 3H, O-CH_3), 1.33-1.29 (t, J = 7.2 Hz, 3H); ^{13}C NMR (50 MHz, DMSO-d_6): δ 158.05, 147.48, 147.29, 143.72, 131.53, 128.09, 123.09, 113.67, 107.55, 97.00, 96.20, 54.97, 44.95, 38.51, 15.66; MS (ES): m/z = 282.4 (M+1); HRMS (ESI): calculated for $C_{17}H_{20}N_3O$ $(M+H)^+$ 282.1606; found 282.1609.

***N*-Butyl-1-ethyl-1*H*-pyrrolo[2,3-*b*]pyridin-4-amine (5e).** Brown solid; mp 93-95 °C; IR (KBr): 3234, 2927, 1872, 1604, 1568, 1512, 1340, 1244, 1035, 819, 799, 617 cm^{-1}; ^{1}H NMR (400 MHz, DMSO-d_6): δ 7.82 (d, J = 5.2 Hz, 1H), 7.13 (d, J = 3.6 Hz, 1H), 6.55 (d, J = 3.2 Hz, 1H), 6.48 (t, J = 5.6 Hz, 1H, NH), 6.11 (d, J = 5.6 Hz, 1H), 4.17 (q, J = 7.2 Hz, 2H), 3.22 (q, J = 6.8 Hz, 2H), 1.61-1.55 (m, 2H), 1.41 (q, J = 7.2 Hz, 2H), 1.33 (t, J = 7.2 Hz, 3H), 0.94 (t, J = 7.2 Hz, 3H); ^{13}C NMR (50 MHz, DMSO-d_6): δ 147.71, 147.32, 143.86, 122.80, 107.35, 97.06, 95.50, 41.79, 38.48, 30.82, 19.77, 15.65, 13.76; MS (ES): m/z = 218.5 (M+1); HRMS (ESI): calculated for $C_{13}H_{20}N_3$ $(M+H)^+$ 218.1657; found 218.1658.

***tert*-Butyl 4-(1-ethyl-1*H*-pyrrolo[2,3-*b*]pyridin-4-yl)piperazine-1-carboxylate (5f).** Brick red solid; mp 82-84 °C; IR (KBr): 2976, 1693, 1573, 1498, 1417, 1365, 1240, 1168, 1001, 756, 663 cm^{-1}; ^{1}H NMR (400 MHz, DMSO-d_6): δ 7.99 (d, J = 5.6 Hz, 1H), 7.36 (d, J = 3.2 Hz, 1H), 6.51 (d, J = 3.6 Hz, 1H), 6.45 (d, J = 5.6 Hz, 1H), 4.24 (q, J = 7.6 Hz, 2H), 3.53 (t, J = 4.8 Hz, 4H), 3.38 (t, J = 4.8 Hz, 4H), 1.43 (s, 9H, C-$(CH_3)_3$), 1.35 (t, J = 7.6 Hz, 3H); ^{13}C NMR (50 MHz, DMSO-d_6): δ 153.79, 150.54, 148.14,

143.36, 124.75, 110.04, 101.47, 98.63, 78.95, 48.45, 43.08, 28.03, 15.50; MS (ES): m/z = 331.5 (M+1); HRMS (ESI): calculated for $C_{18}H_{27}N_4O_2$ $(M+H)^+$ 331.2134; found 331.2139.

General procedure for C-N-bond formation by coupling of 4-bromo-7-azaindole derivatives with amino acid ester

To a 100 mL dried sealed Schlenk tube, N-substituted 4-bromo azaindole (1.0 mmol), amino acids/esters (1.2 mmol), cesium carbonate (3.0 mmol), $Pd_2(dba)_3$ (5 mole %), Xantphos (10 mole %), and 2 mL of dioxane were added. Nitrogen gas was bubbled through the reaction mass for 10 minutes. The reaction mixture was heated to 100 °C and stirred for an appropriate time as mentioned in Table 6. The reaction mass was cooled to room temperature and diluted with ethyl acetate (20 mL), filtered through a celite bed and wash with ethyl acetate (10 mL).The filtrate was concentrated in vacuum. The crude product was purified by column chromatography on silica gel (100-200) using a ethyl acetate and hexane mixture as an eluent to afford the pure title products.

(*R*)-methyl 2-((1-methyl-1*H*-pyrrolo[2,3-*b*]pyridin-4-yl)amino)propanoate (7b). Pale yellow solid; ^{1}H NMR (400 MHz, $CDCl_3$): δ 8.08 (d, *J* = 5.2 Hz, 1H), 6.98 (d, *J* = 3.6 Hz, 1H), 6.38 (d, *J* = 3.6 Hz, 1H), 6.15 (d, *J* = 5.2 Hz, 1H), 4.94 (d, *J* = 8.0 Hz, 1H), 4.42-4.35 (m, 1H), 3.83 (s, 3H), 3.77 (s, 3H), 1.58 (d, *J* = 7.2 Hz, 3H); ^{13}C NMR (100 MHz, $CDCl_3$): δ 174.19, 148.30, 146.01, 144.53, 125.53, 108.13, 96.80, 95.60, 52.43, 50.97, 31.42, 18.75; MS (ES): m/z = 234.20 (M+1); HRMS (ESI): calculated for $C_{12}H_{15}N_3O_2$ $(M+H)^+$ 234.1234; found 234.1236; ee% (the methyl ester) 98.79 (HPLC: Chiral Pak AD-H Column, n-heptane:ethanol:IP amine (60:40:0.10), 1.0 mL/min, 240nm, t = 30 °C).

Ethyl 2-((1-methyl-1*H*-pyrrolo[2,3-*b*]pyridin-4-yl)amino)acetate (7c). Pale yellow solid; ^{1}H NMR (400 MHz, $CDCl_3$): δ 8.10 (d, *J* = 5.6 Hz, 1H), 6.98 (d, *J* = 3.6 Hz, 1H), 6.40 (d, *J* = 3.6 Hz, 1H), 6.13 (d, *J* = 5.6 Hz, 1H), 5.02 (s, 1H), 4.31-4.26 (m, 2H), 4.09 (d, *J* = 4.8 Hz, 2H), 3.83 (s, 3H), 1.34-1.30 (t, *J* = 7.2 Hz, 3H); ^{13}C NMR (100 MHz, $CDCl_3$): δ 170.35, 148.22, 146.48, 144.54, 125.48, 107.99, 96.73, 95.64, 61.54, 44.74, 31.38, 14.09; MS (ES): m/z = 234.20 (M+1); HRMS (ESI): calculated for $C_{12}H_{16}N_3O_2$ $(M+H)^+$ 234.1243; found 234.1237.

(*R*)-methyl 3-(*tert*-butoxy)-2-((1-methyl-1*H*-pyrrolo[2,3-*b*]pyridin-4-yl) amino)propanoate (7d). Pale yellow solid; ^{1}H NMR (400 MHz, $CDCl_3$): δ 8.07 (d, *J* = 5.2 Hz, 1H), 6.98 (d, *J* = 3.2 Hz, 1H), 6.40 (d, *J* = 3.6 Hz, 1H), 6.14 (d, *J* = 5.2 Hz, 1H), 5.23 (d, *J* = 8.8 Hz, 1H), 4.4-4.40 (m, 1H), 3.88 (dd, *J* = 4.0 & 8.8 Hz, 1H), 3.83 (s, 3H), 3.77 (m, 1H), 3.76 (s, 3H), 1.17 (s, 9H); ^{13}C NMR (100 MHz, $CDCl_3$): δ 171.95, 148.38, 146.46, 144.47, 125.50, 108.34, 97.02, 95.74, 73.67, 62.19, 56.08, 52.34, 31.46, 27.30; MS (ES): m/z = 306.20 (M+1); HRMS (ESI): calculated for $C_{16}H_{24}N_3O_3$ $(M+H)^+$ 361.1818; found 361.1815; ee% (the methyl ester) 95.48 (HPLC: Chiral Pak AD-H Column, n-heptane:ethanol:IP amine (60:40:0.10), 1.0 mL/min, 240nm, t = 30 °C).

(*R*)-methyl 2-((1-methyl-1*H*-pyrrolo[2,3-*b*]pyridin-4-yl)amino)propanoate (7e). Pale yellow solid; ^{1}H NMR (400 MHz, $CDCl_3$): δ 8.07 (d, *J* = 6.0 Hz, 1H), 7.04 (d, *J* = 3.6 Hz, 1H), 6.39 (d, *J* = 3.6 Hz, 1H), 6.15 (d, *J* = 5.2 Hz, 1H), 4.91 (d, *J* = 8.0 Hz, 1H), 4.40-4.35 (m, 1H), 4.31-4.25 (m, 2H), 3.77 (s, 3H), 1.58 (d, *J* = 6.8 Hz, 3H), 1.47 (t, *J* = 7.0 Hz, 3H); ^{13}C NMR (100 MHz, $CDCl_3$): δ 174.20, 147.68, 145.97, 144.39, 123.79, 108.21, 96.76, 95.65, 52.39, 50.94, 39.36, 18.74, 15.68; MS (ES): m/z = 248.20 (M+1); HRMS (ESI): calculated for $C_{13}H_{18}N_3O_2$ $(M+H)^+$ 248.1399; found

248.1398; ee% (the methyl ester) 98.91 (HPLC: Chiral Pak AD-H Column, n-heptane:ethanol:IP amine (60:40:0.10), 1.0 mL/min, 240nm, t = 30 °C).

Ethyl 2-((1-ethyl-1*H*-pyrrolo[2,3-*b*]pyridin-4-yl)amino)acetate (7f). Pale yellow solid; ^{1}H NMR (400 MHz, $CDCl_3$): δ 8.09 (d, *J* = 5.6 Hz, 1H), 7.04 (d, *J* = 3.6 Hz, 1H), 6.40 (d, *J* = 3.2 Hz, 1H), 6.12 (d, *J* = 5.2 Hz, 1H), 5.08 (s, 1H), 4.31-4.26 (m, 4H), 4.09 (d, *J* = 4.8 Hz, 2H), 1.47-1.43 (t, *J* = 7.2 Hz, 3H), 1.34-1.31 (t, *J* = 6.8 Hz, 3H); ^{13}C NMR (100 MHz, $CDCl_3$): δ 170.37, 147.62, 146.47, 144.42, 123.75, 108.11, 96.72, 95.69, 61.53, 44.75, 39.33, 15.68, 14.09; MS (ES): m/z = 248.20 (M+1); HRMS (ESI): calculated for $C_{13}H_{18}N_3O_2$ $(M+H)^+$ 248.1399; found 248.1397.

Methyl 2-((1-ethyl-1*H*-pyrrolo[2,3-*b*]pyridin-4-yl)amino)acetate (7g). Pale yellow solid; ^{1}H NMR (400 MHz, $CDCl_3$): δ 8.09 (d, *J* = 5.2 Hz, 1H), 7.04 (d, *J* = 3.2 Hz, 1H), 6.41 (d, *J* = 4.0 Hz, 1H), 6.12 (d, *J* = 5.2 Hz, 1H), 4.99 (s, 1H), 4.31-4.26 (m, 2H), 4.11 (d, *J* = 5.6 Hz, 2H), 3.83 (s, 3H), 1.47-1.44 (t, *J* = 7.2 Hz, 3H); ^{13}C NMR (100 MHz, $CDCl_3$): δ 170.84, 147.58, 146.42, 144.38, 123.78, 108.09, 96.65, 95.67, 52.33, 44.55, 39.32, 15.65; MS (ES): m/z = 234.20 (M+1); HRMS (ESI): calculated for $C_{12}H_{16}N_3O_2$ $(M+H)^+$ 234.1243; found 234.1236.

General procedure for C–O bond formation by coupling of 4-bromo-7-azaindole derivatives with phenols

To a 100 mL dried sealed Schlenk tube, N-substituted 4-bromo-azaindole (1.0 mmol), phenol (1.2 mmol), potassium carbonate (1.5 mmol), $Pd(OAc)_2$ (5 mol %), Xantphos (10 mol %), and 2 mL of dioxane were added. Nitrogen was bubbled through the reaction mass for 2 min. The reaction mixture was heated to 100 °C and stirred for an appropriate time as mentioned in Table 8. The reaction mass cooled to room

temperature and was diluted with ethyl acetate (20 mL), filtered through a celite bed and wash with ethyl acetate (10 mL). The filtrate was concentrated in vacuum. The crude product was purified by column chromatography on silica gel (100–200) using ethyl acetate and hexane mixture as an eluent to afford the pure title products.

1-Methyl-4-(*m*-tolyloxy)-1*H*-pyrrolo[2,3-*b*]pyridine (9a). ^{1}H NMR (400 MHz, $CDCl_3$): δ□8.18 (d, *J* = 5.6 Hz, 1H), 7.29-7.25 (m, 2H), 7.05-6.91 (m, 3H), 6.48 (d, *J* = 5.6 Hz, 1H), 6.32 (d, *J* = 3.6 Hz, 1H), 3.88 (s, 3H, N-CH_3), 2.36 (s, 3H, -CH_3); ^{13}C NMR (100 MHz, $CDCl_3$): δ 158.11, 155.17, 144.33, 140.04, 129.48, 127.40, 125.46, 121.04, 117.39, 111.36, 102.81, 97.02, 31.53, 21.35; MS (ES): m/z = 239.10 (M+1); HRMS (ESI): calculated for $C_{15}H_{15}N_2O$ $(M+H)^+$ 239.1184; found 239.1174.

4-(4-Methoxyphenoxy)-1-methyl-1*H*-pyrrolo[2,3-*b*]pyridine (9b). ^{1}H NMR (400 MHz, $CDCl_3$): δ 8.15 (d, *J* = 5.2 Hz, 1H), 7.12-7.07 (m, 2H), 7.05 (d, *J* = 3.6 Hz, 1H), 6.95-6.92 (m, 2H), 6.40 (d, *J* = 5.2 Hz, 1H), 6.33 (d, *J* = 3.2 Hz, 1H), 3.88 (s, 3H, N-CH3), 3.84 (s, 3H, O-CH_3); ^{13}C NMR (100 MHz, $CDCl_3$): δ 159.03, 156.75, 148.36, 144.23, 127.34, 121.84, 116.13, 114.85, 110.99, 101.91, 97.05, 55.63, 31.62; MS (ES): m/z = 255.10 (M+1); HRMS (ESI): calculated for $C_{15}H_{15}N_2O_2$ $(M+H)^+$ 255.1134; found 255.1144.

1-Methyl-4-(naphthalen-1-yloxy)-1*H*-pyrrolo[2,3-*b*]pyridine (9c). ^{1}H NMR (400 MHz, $CDCl_3$): δ 8.14 (d, *J* = 5.6 Hz, 1H), 8.07 (d, *J* = 8.4, 1H)), 7.92 (d, *J* = 8.4 Hz, 1H), 7.76 (d, *J* = 8.4 Hz, 1H), 7.55-7.44 (m, 4H), 7.23 (t, *J* = 7.6 Hz, 1H), 7.08 (d, *J* = 3.6 Hz, 1H), 6.39 (d, *J* = 3.6 Hz, 1H), 3.90 (s, 3H, N-CH_3; ^{13}C NMR (100 MHz, $CDCl_3$): δ 158.69, 150.85, 144.43, 134.96, 127.89, 127.55, 128.08, 126.67, 126.29, 125.68, 125.01, 121.99, 116.26, 111.07, 102.55, 102.44, 96.99, 31.60; MS (ES): m/z = 275.10 (M+1); HRMS (ESI): calculated for $C_{18}H_{15}N_2O$ $(M+H)^+$ 275.1184; found 275.1175

^{1}H NMR of **3a** in DMSO-d_6, 400 MHz

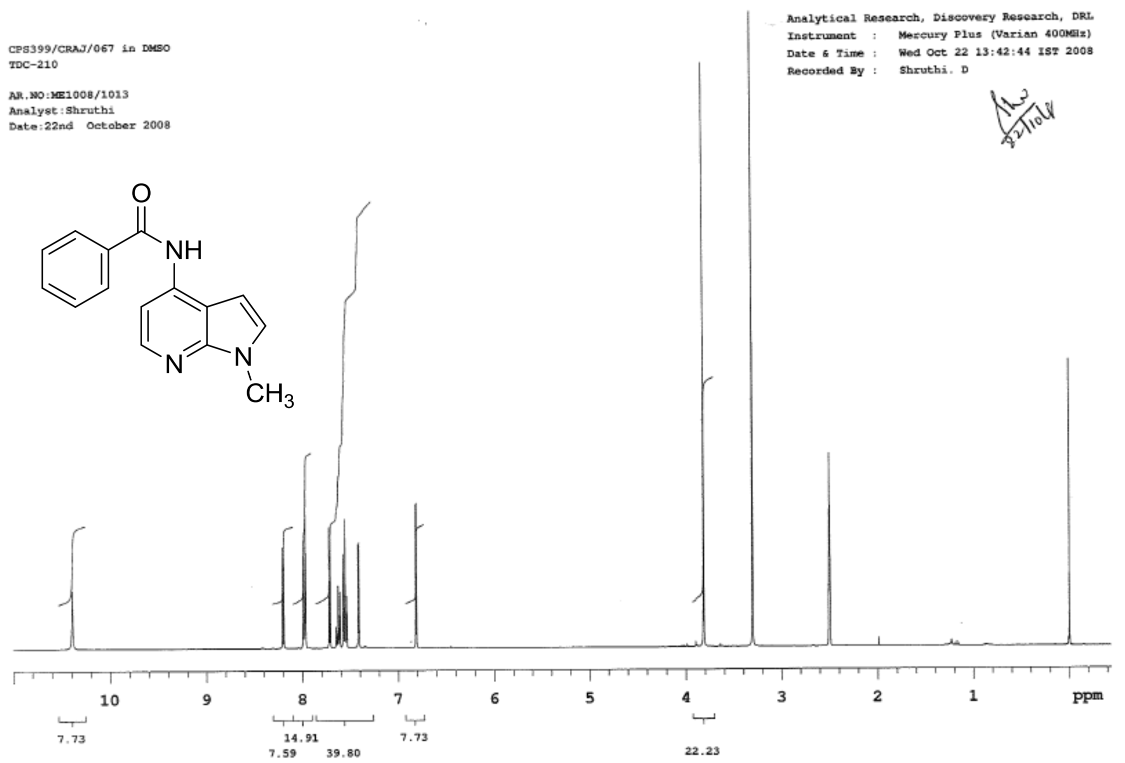

^{13}C NMR of **3a** in DMSO-d_6, 50 MHz.

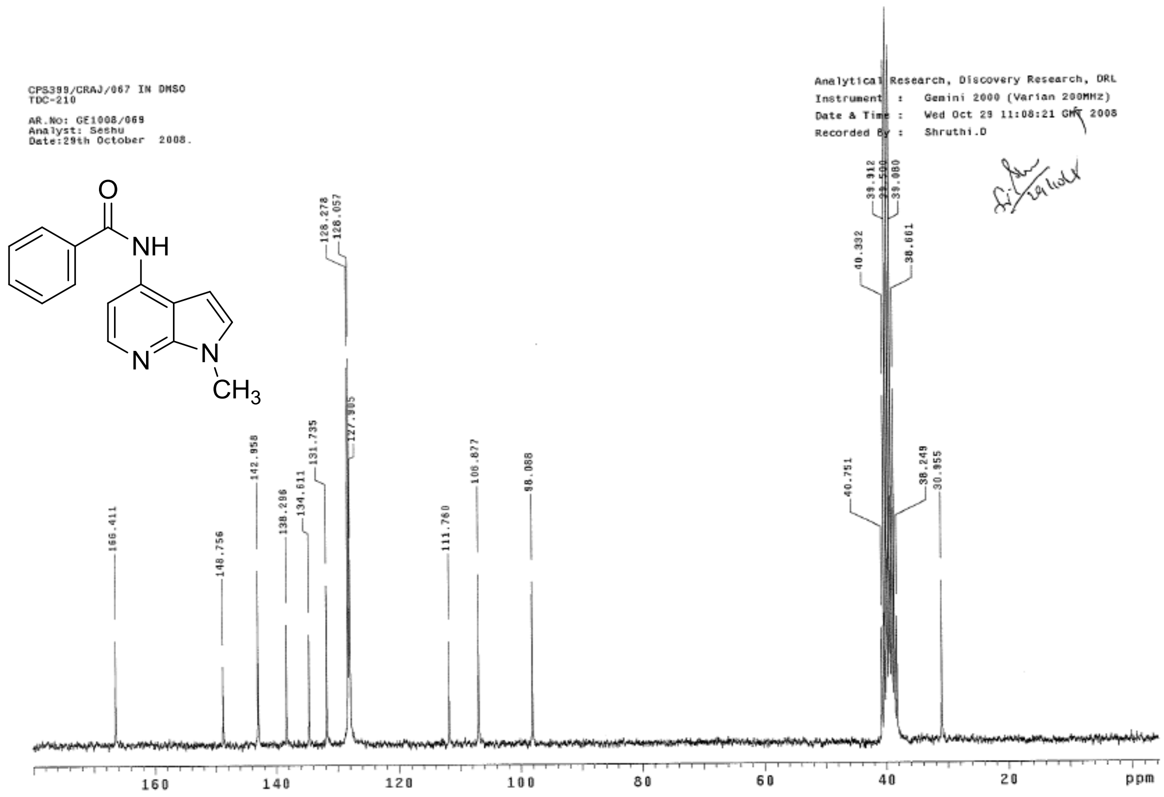

^{1}H NMR of **3b** in DMSO-d_6, 400 MHz.

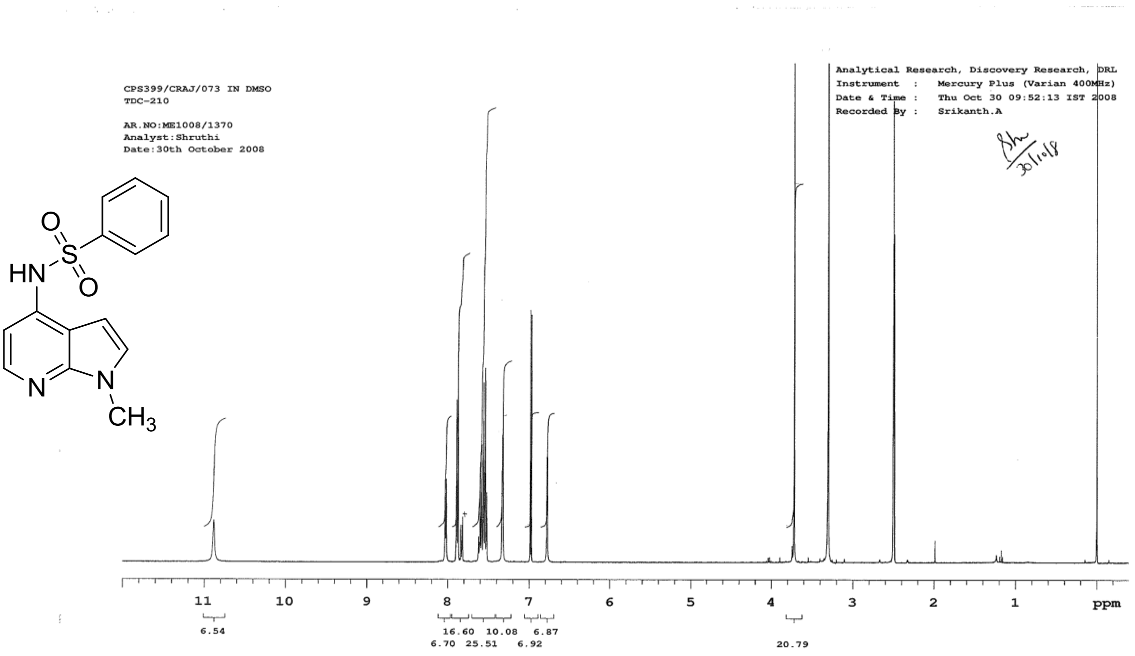

^{13}C NMR of **3b** in DMSO-d_6, 50 MHz.

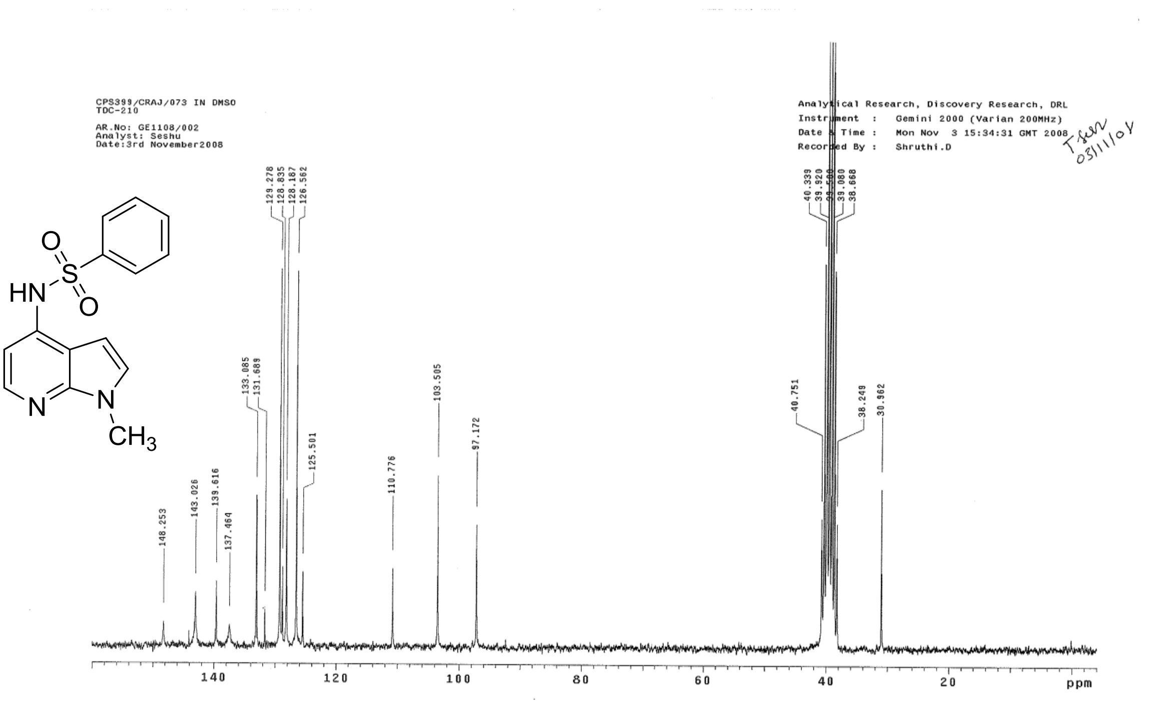

^{1}H NMR of **3c** in DMSO-d_6, 400 MHz.

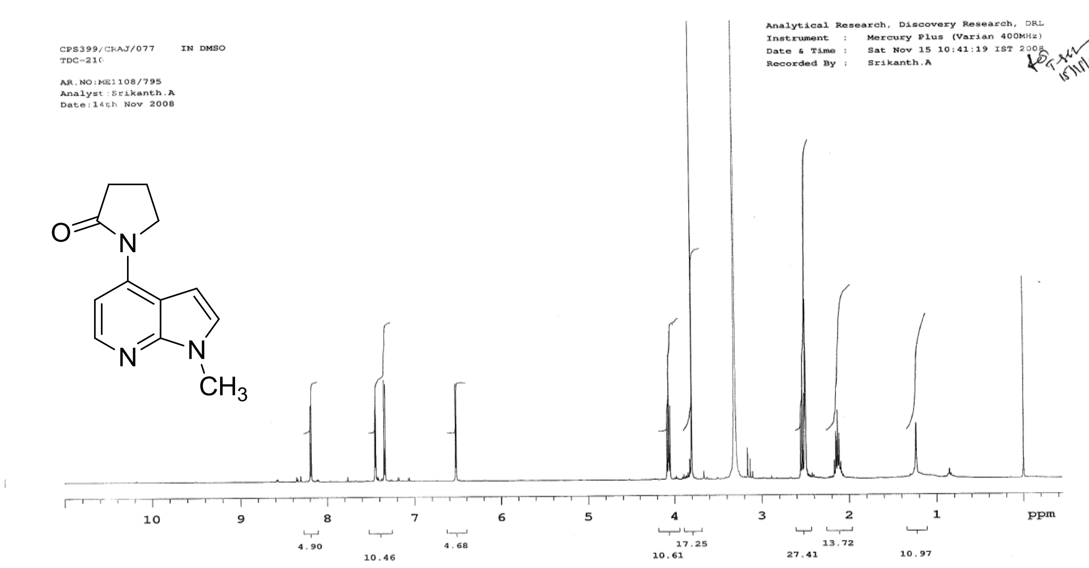

^{13}C NMR of **3c** in DMSO-d_6, 50 MHz.

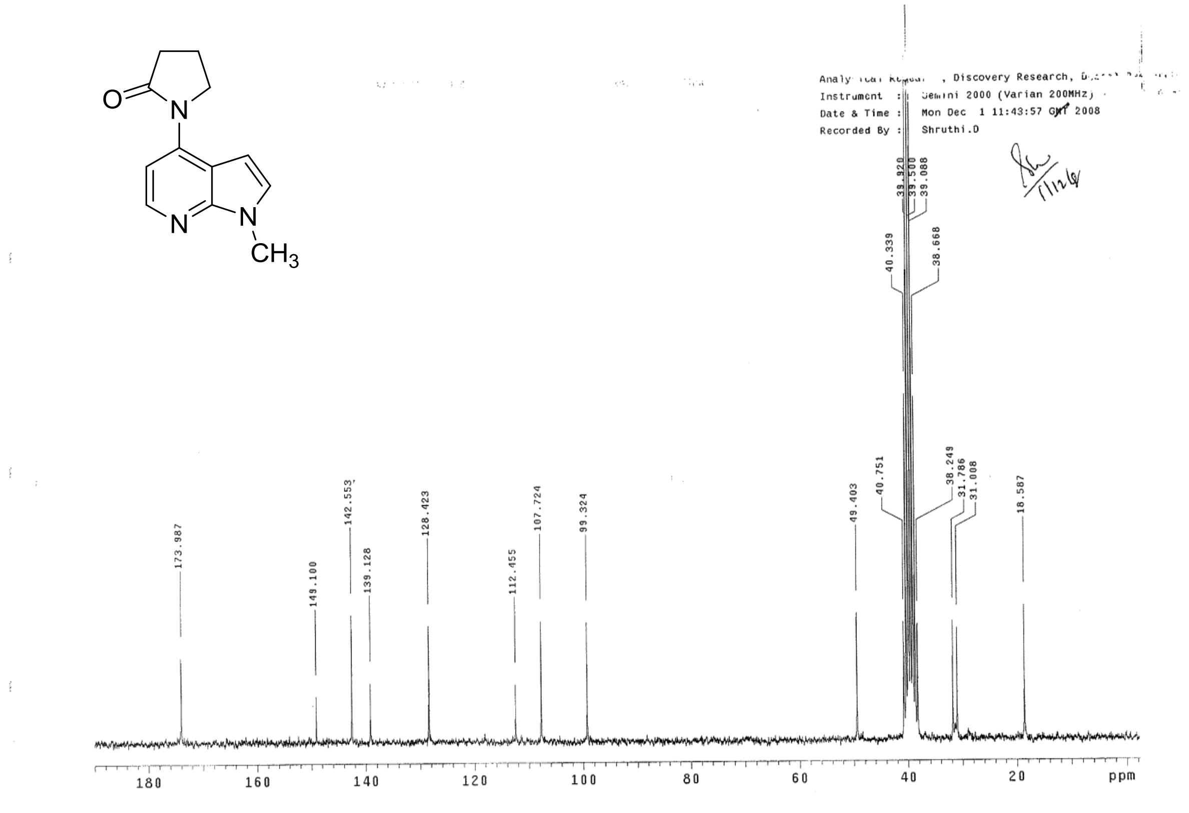

^{1}H NMR of **3d** in DMSO-d_6, 400 MHz.

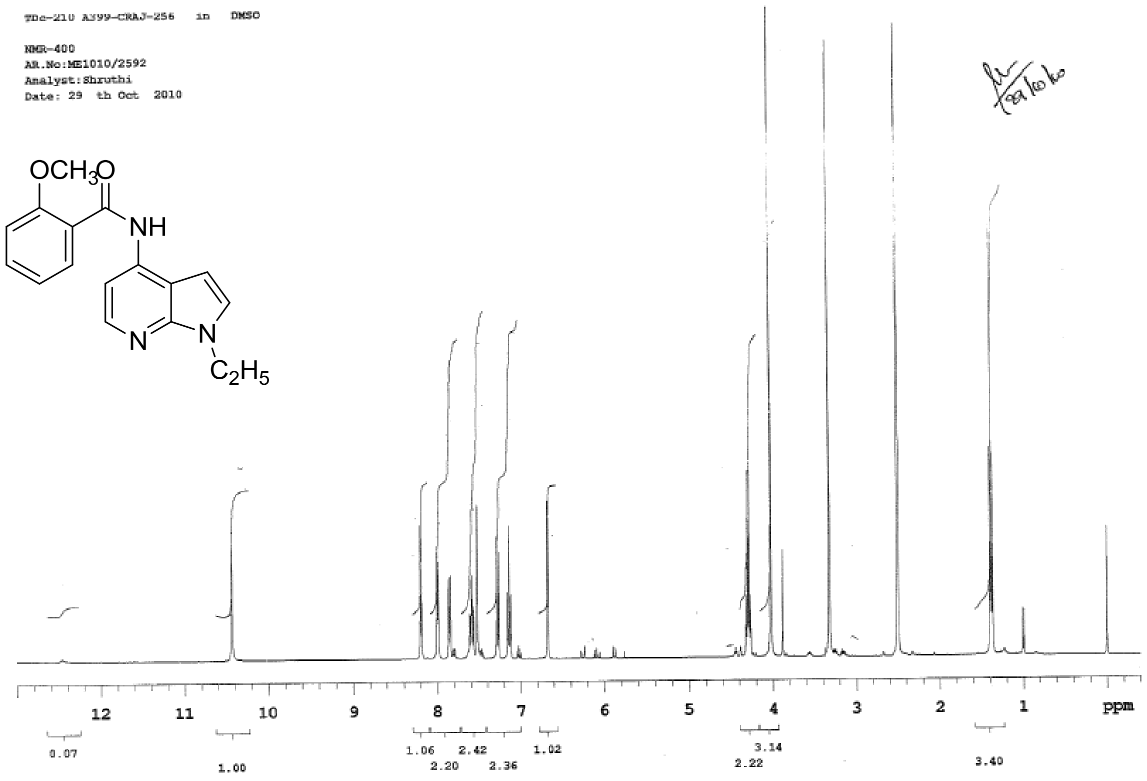

^{13}C NMR of **3d** in DMSO-d_6, 50 MHz.

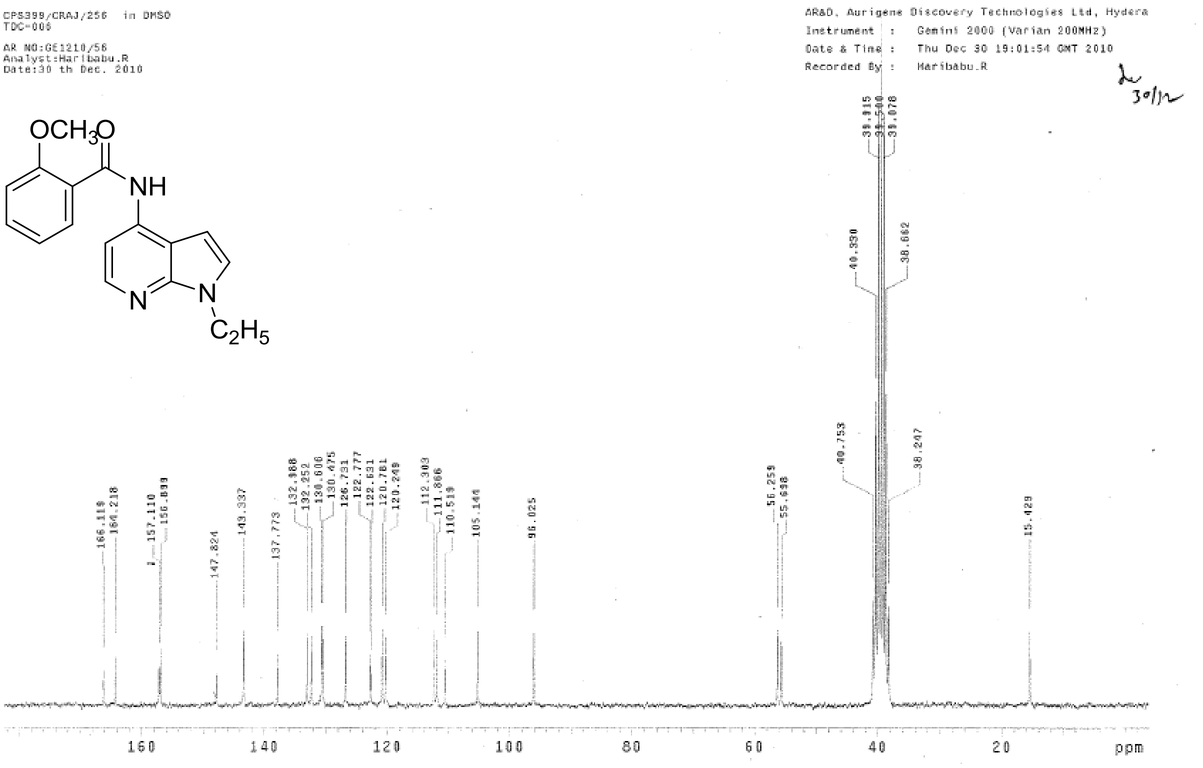

^{1}H NMR of **3e** in DMSO-d_6, 500 MHz.

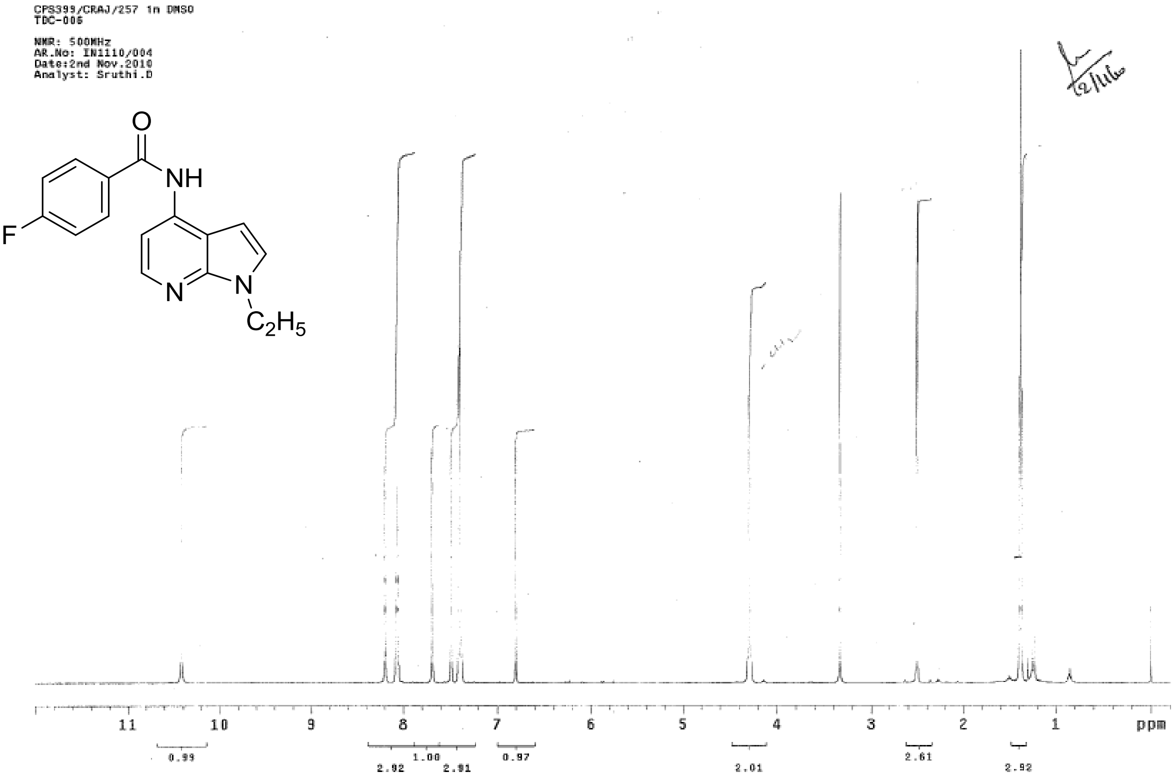

^{13}C NMR of **3e** in DMSO-d_6, 100 MHz.

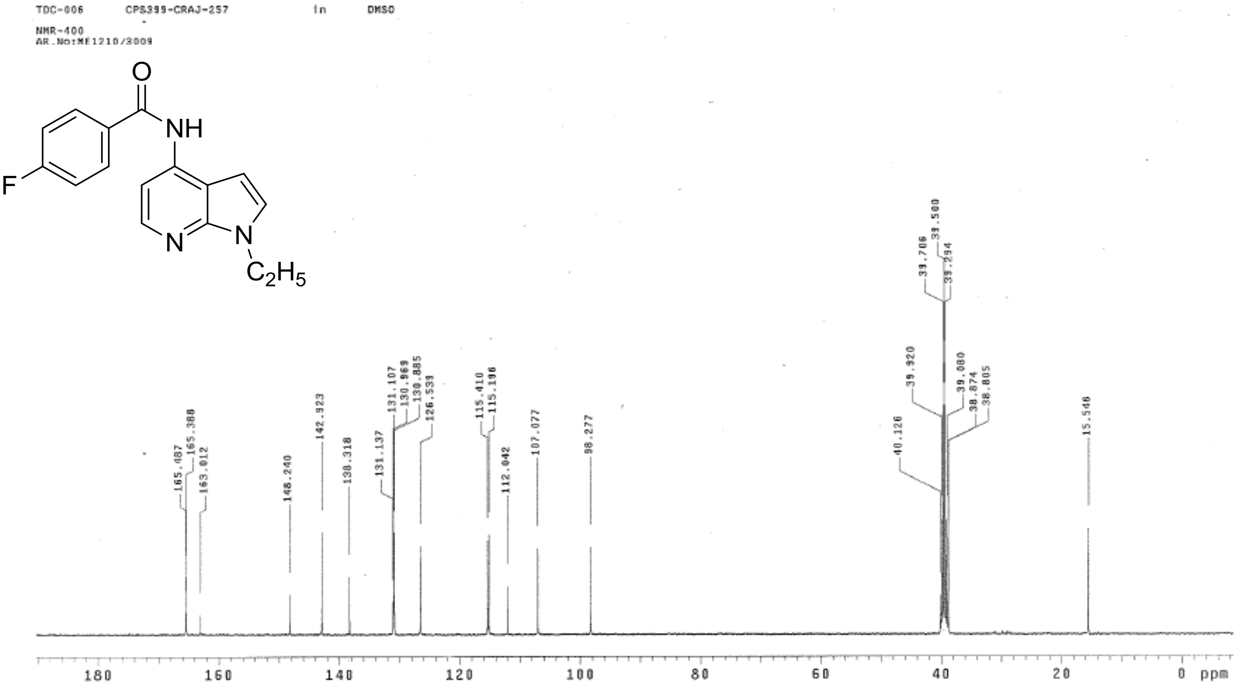

^{1}H NMR of **3f** in DMSO-d_6, 400 MHz.

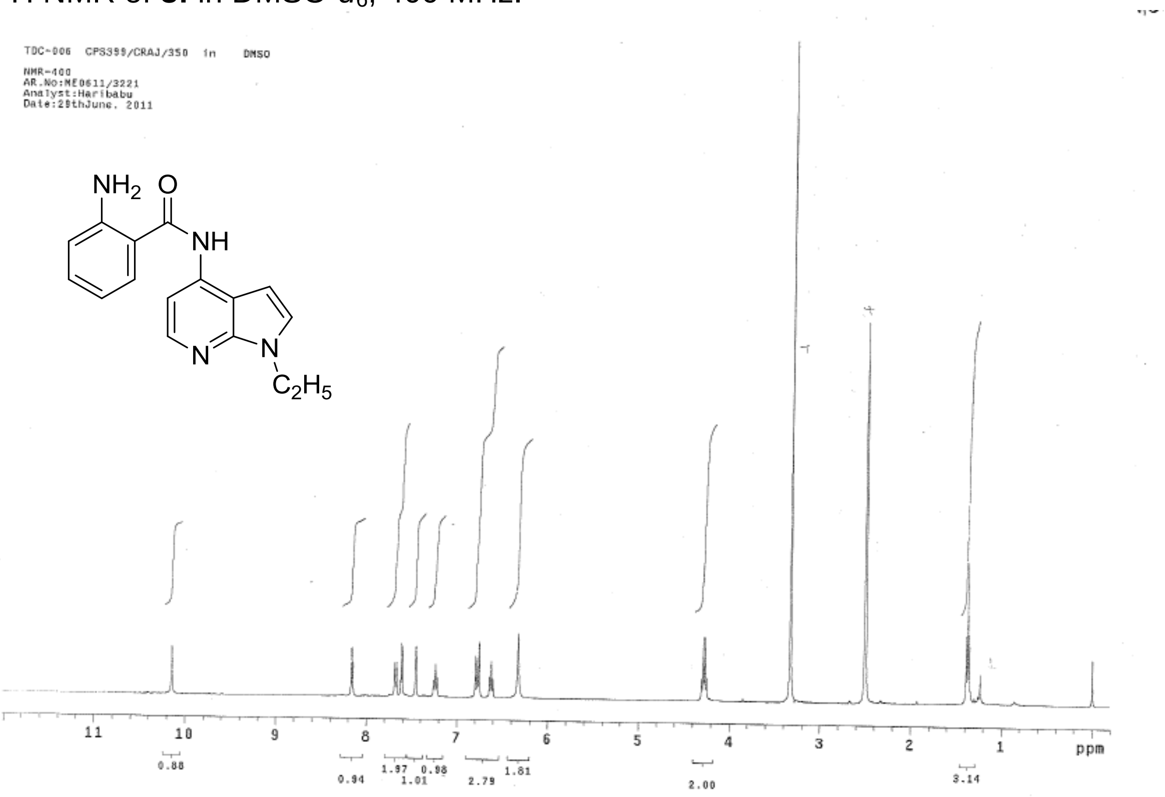

^{13}C NMR of **3f** in DMSO-d_6, 100 MHz.

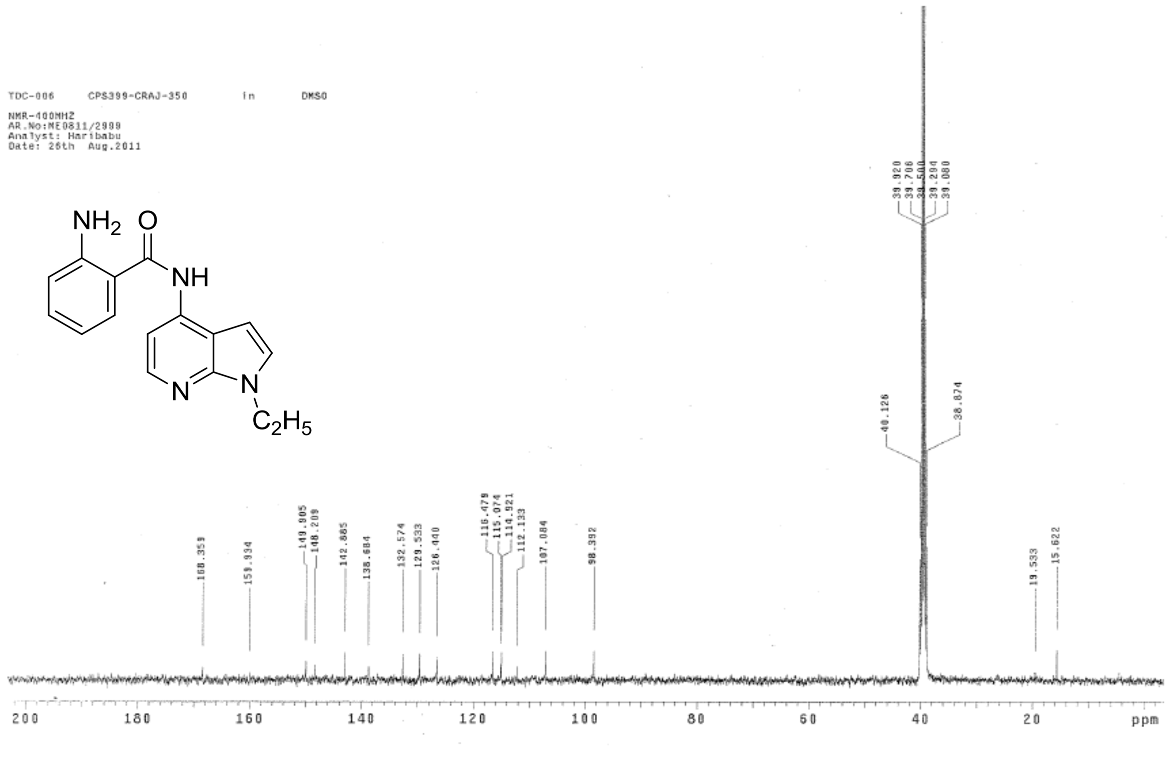

^{1}H NMR of **5a** in DMSO-d_6, 400 MHz.

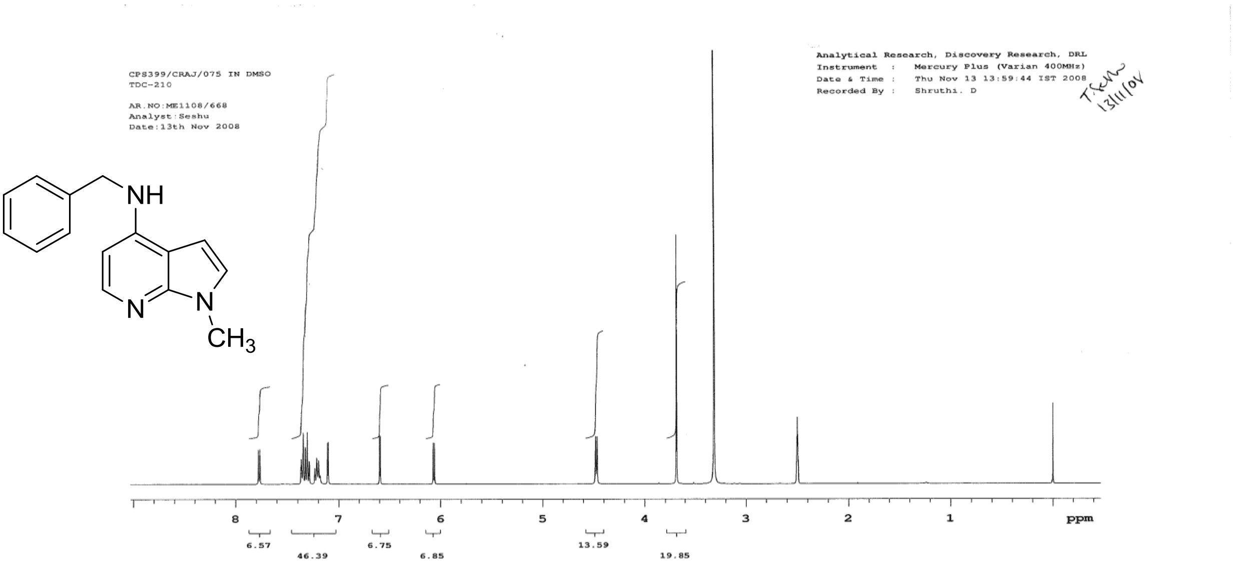

^{13}C NMR of **5a** in DMSO-d_6, 50 MHz.

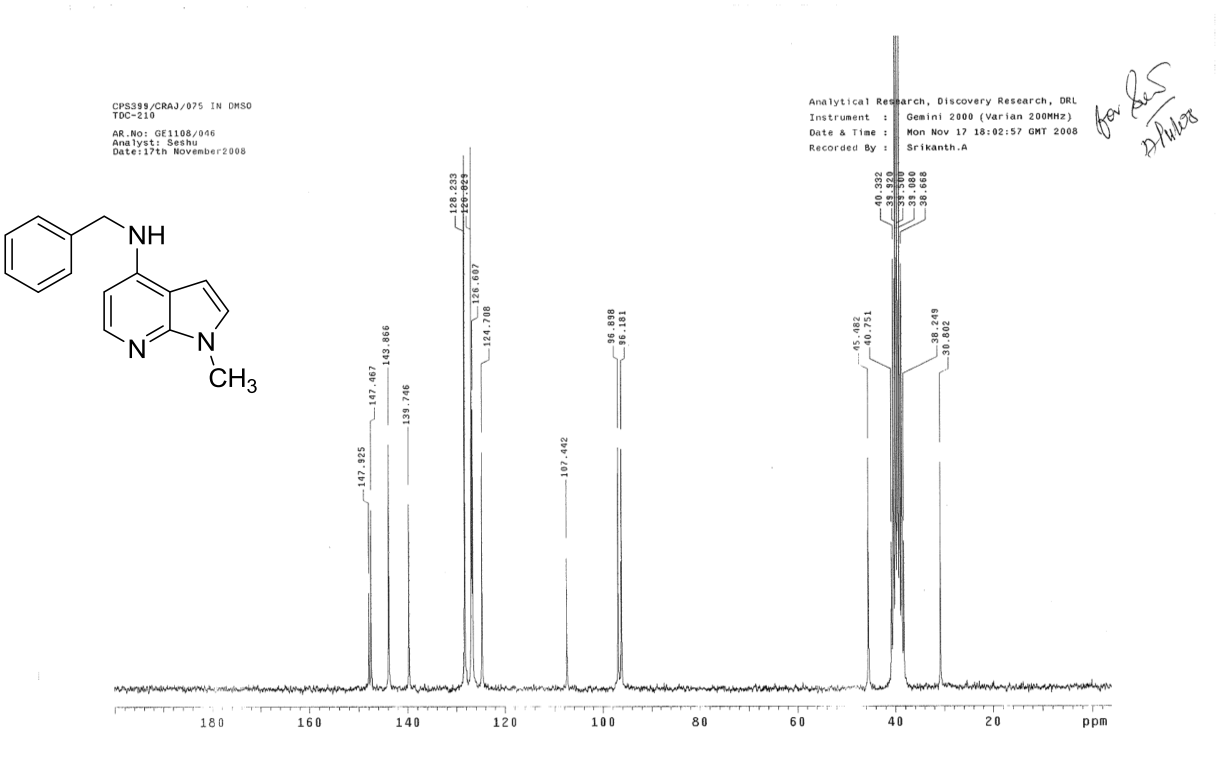

^{1}H NMR of **5b** in DMSO-d_6, 400 MHz.

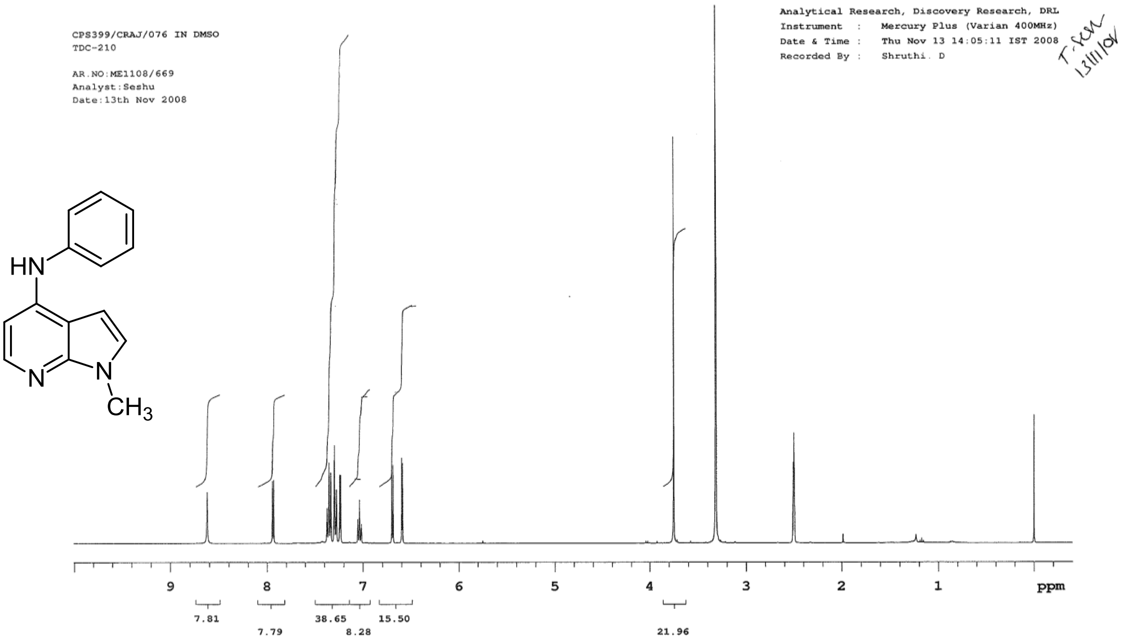

^{13}C NMR of **5b** in DMSO-d_6, 50 MHz.

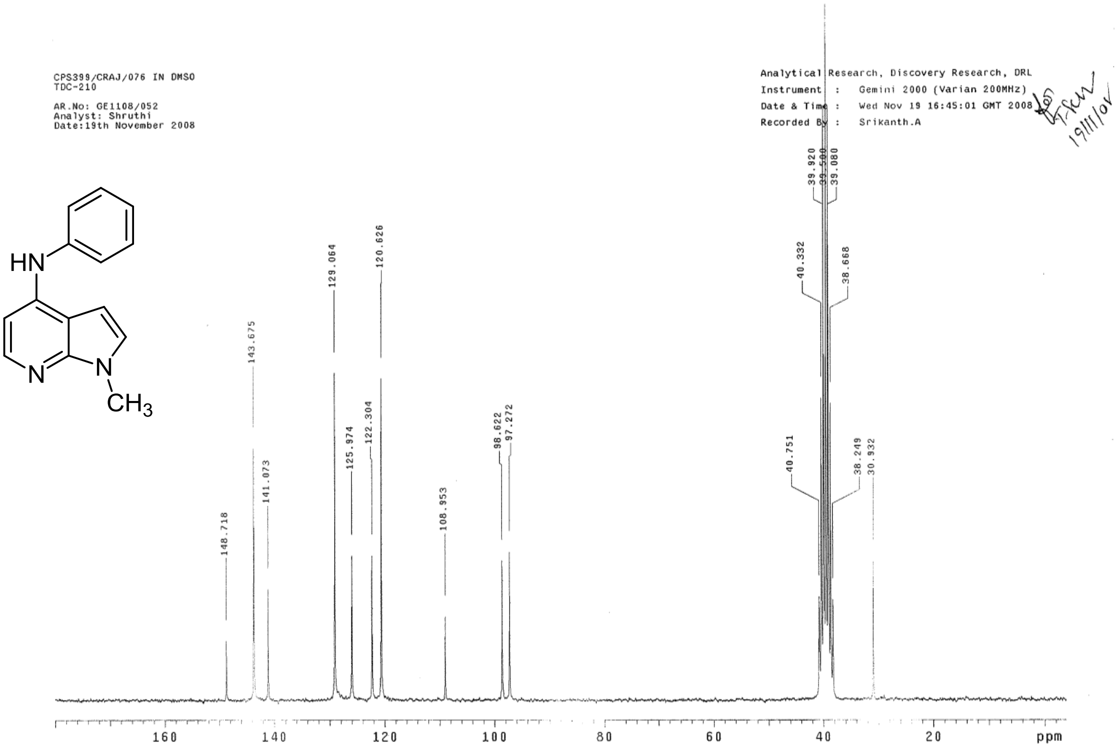

^{1}H NMR of **5c** in DMSO-d_6, 400 MHz.

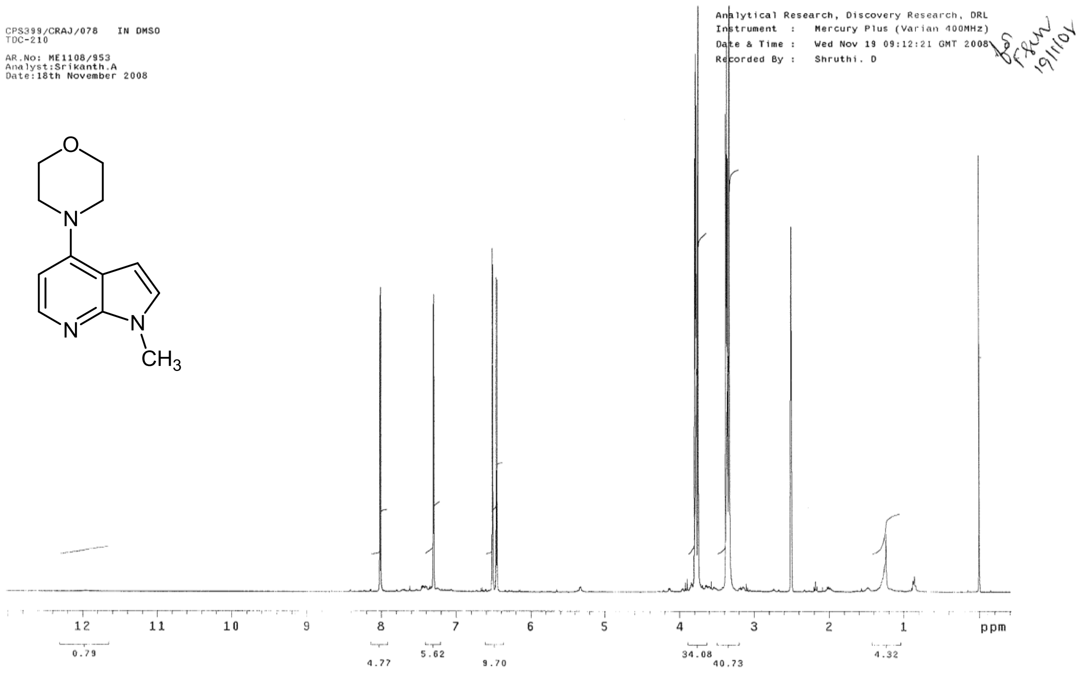

^{13}C NMR of **5c** in DMSO-d_6, 50 MHz.

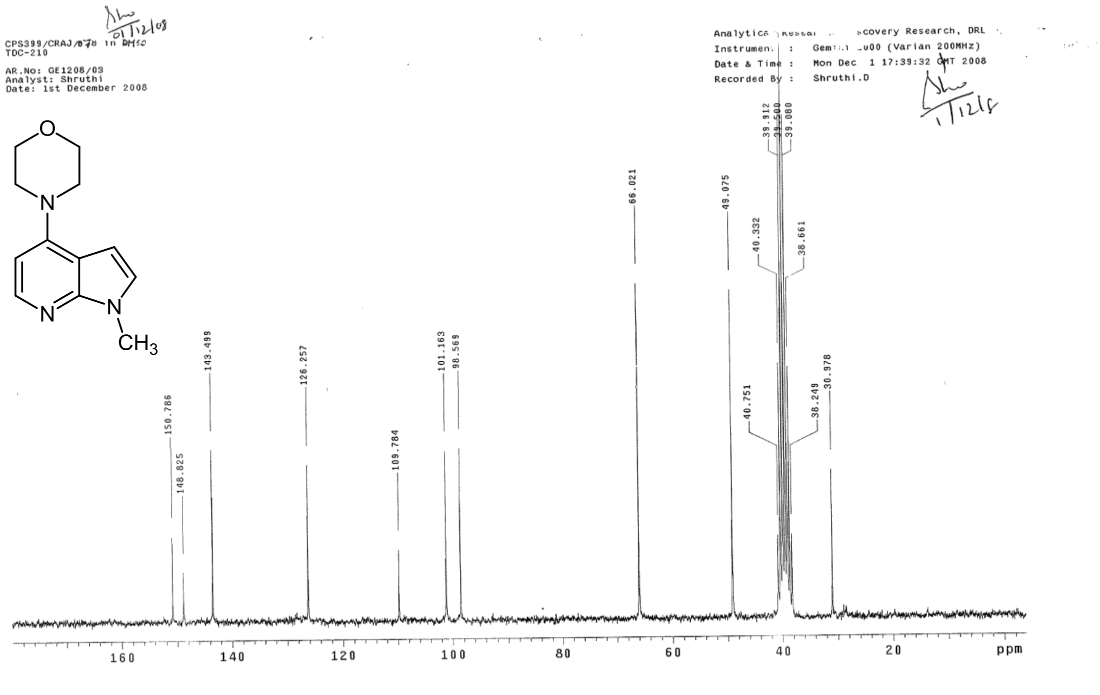

^{1}H NMR of **5d** in DMSO-d_6, 400 MHz.

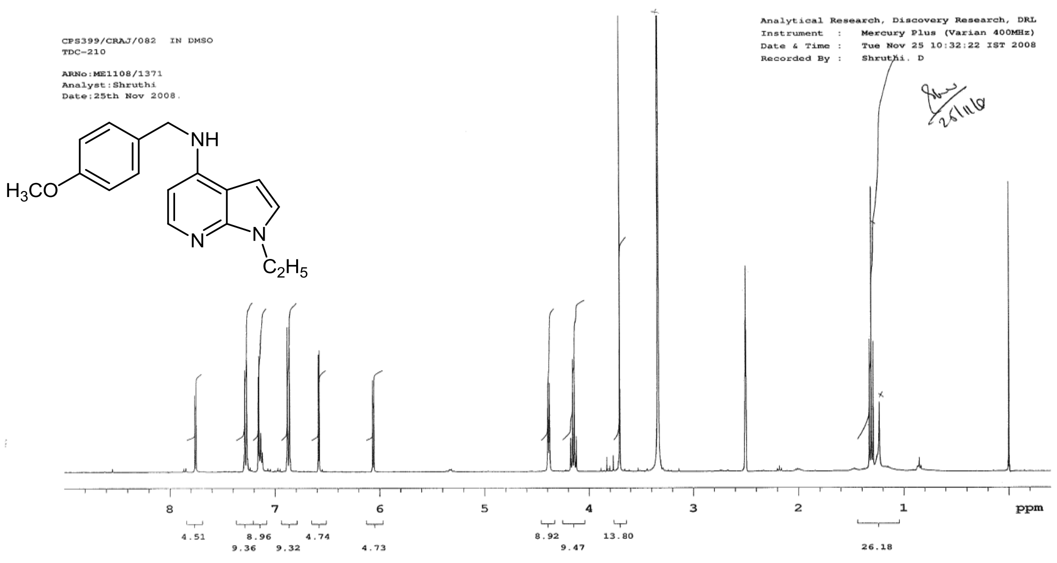

^{13}C NMR of **5d** in DMSO-d_6, 50 MHz.

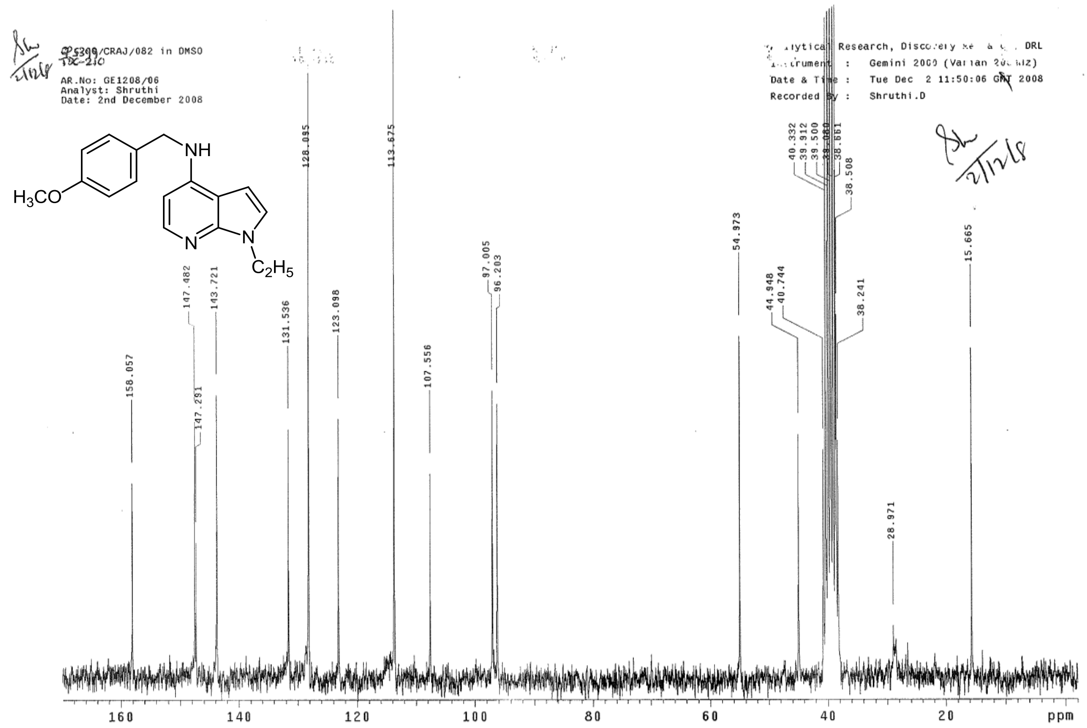

^{1}H NMR of **5e** in DMSO-d_6, 400 MHz.

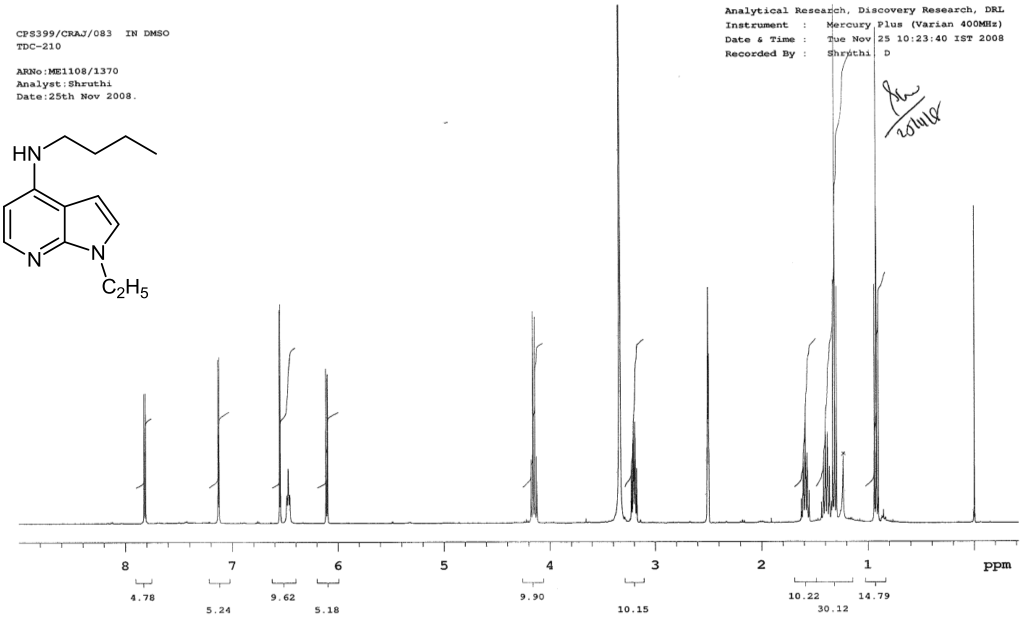

^{13}C NMR of **5e** in DMSO-d_6, 50 MHz.

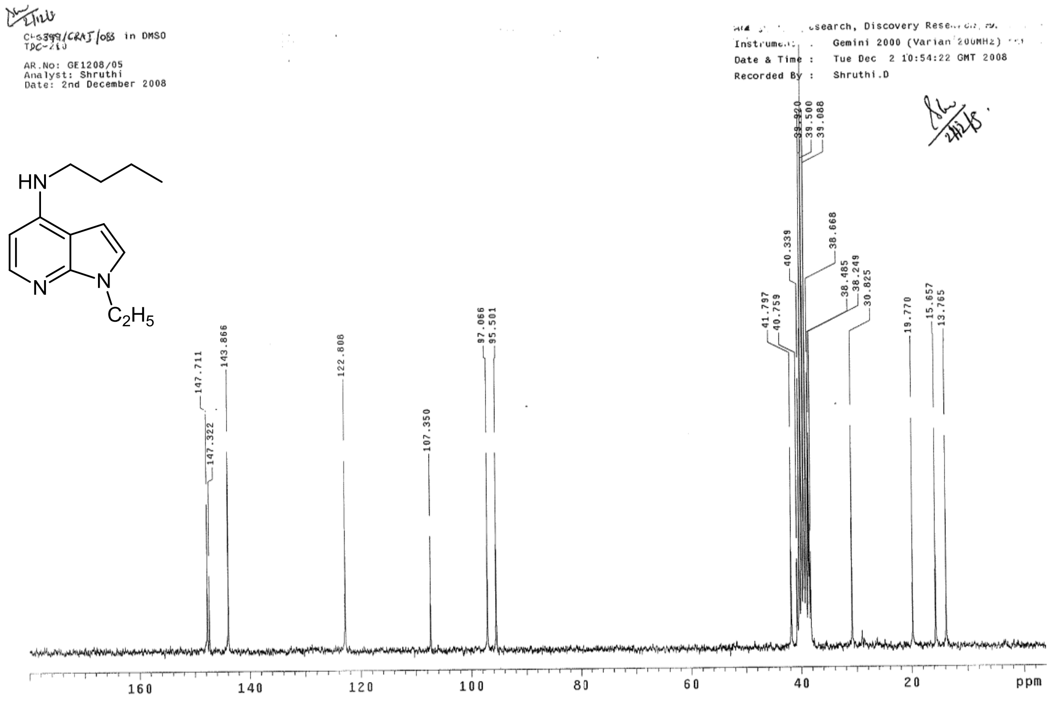

^{1}H NMR of **5f** in DMSO-d_6, 400 MHz.

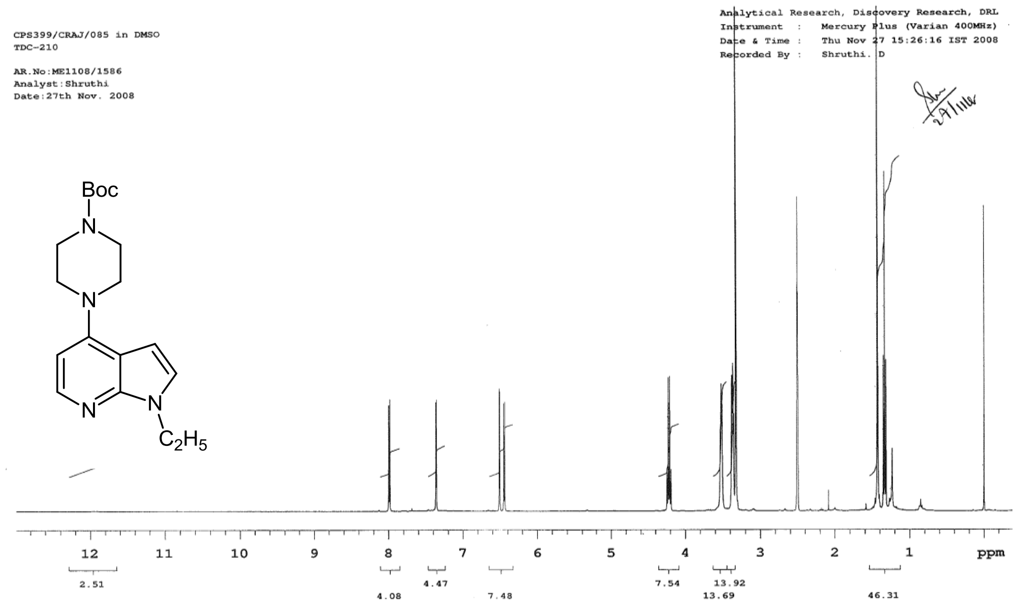

^{13}C NMR of **5f** in DMSO-d_6, 50 MHz.

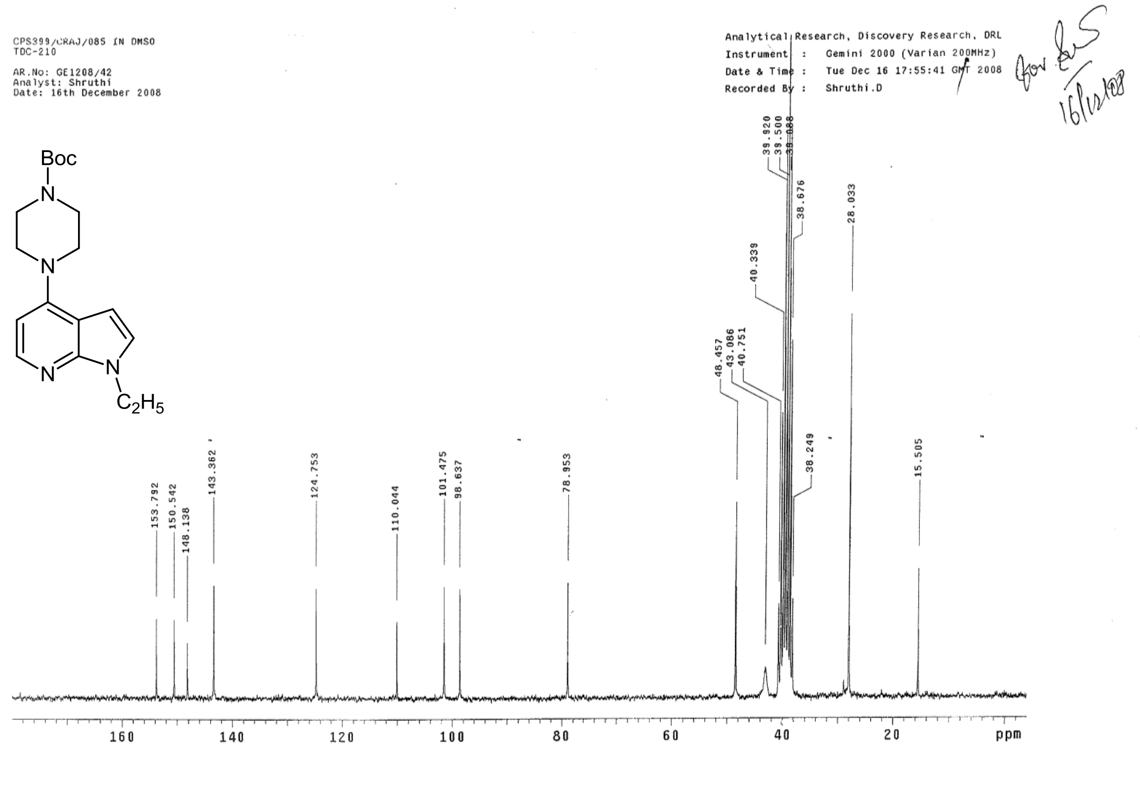

^{1}H NMR of **7b** in DMSO-d_6, 400 MHz.

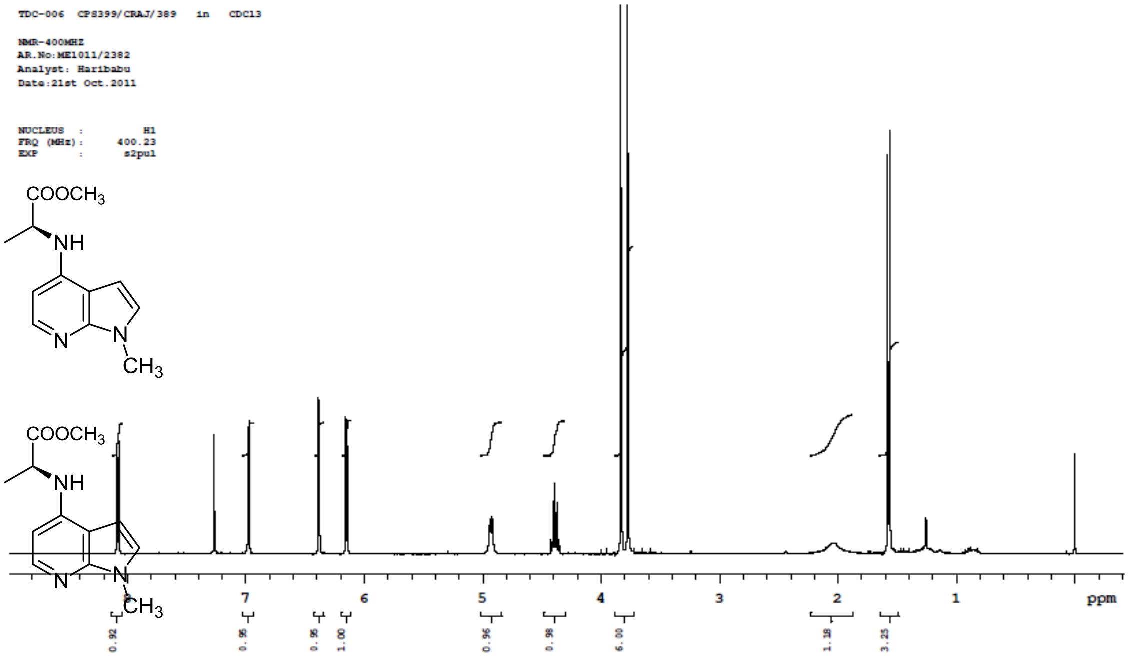

^{13}C NMR of **7b** in DMSO-d_6, 100 MHz.

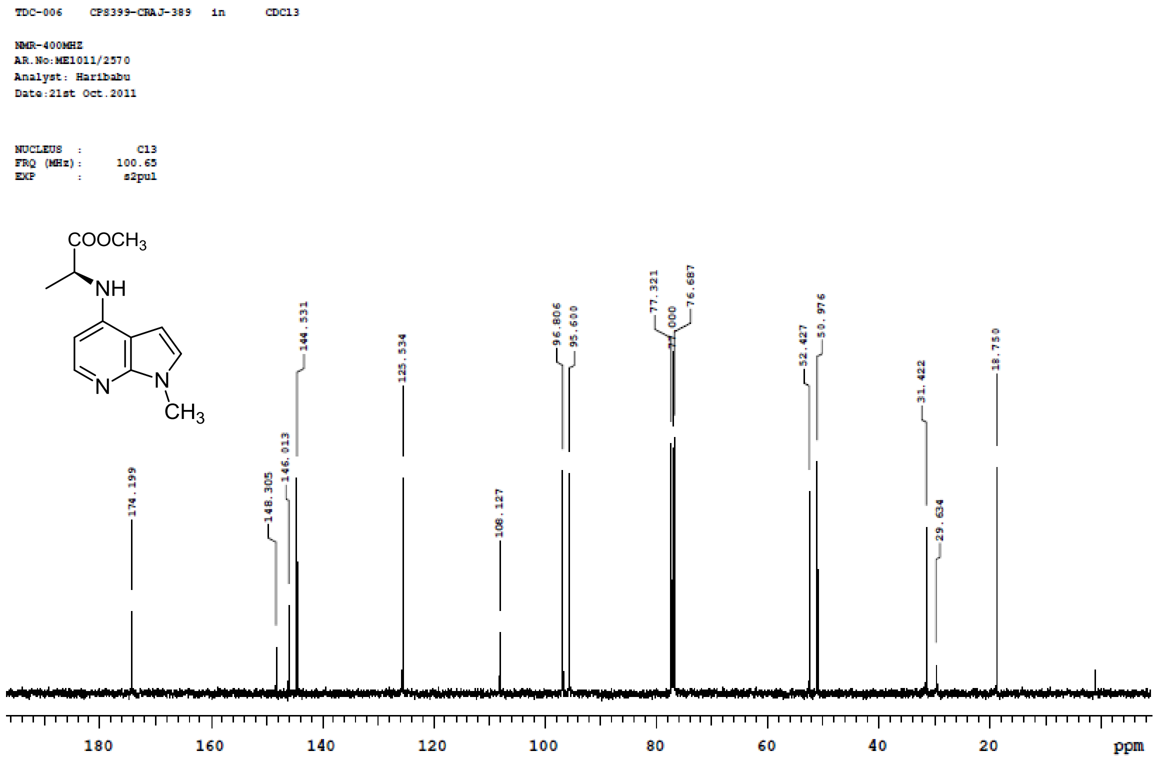

^{1}H NMR of **7c** in DMSO-d_6, 400 MHz.

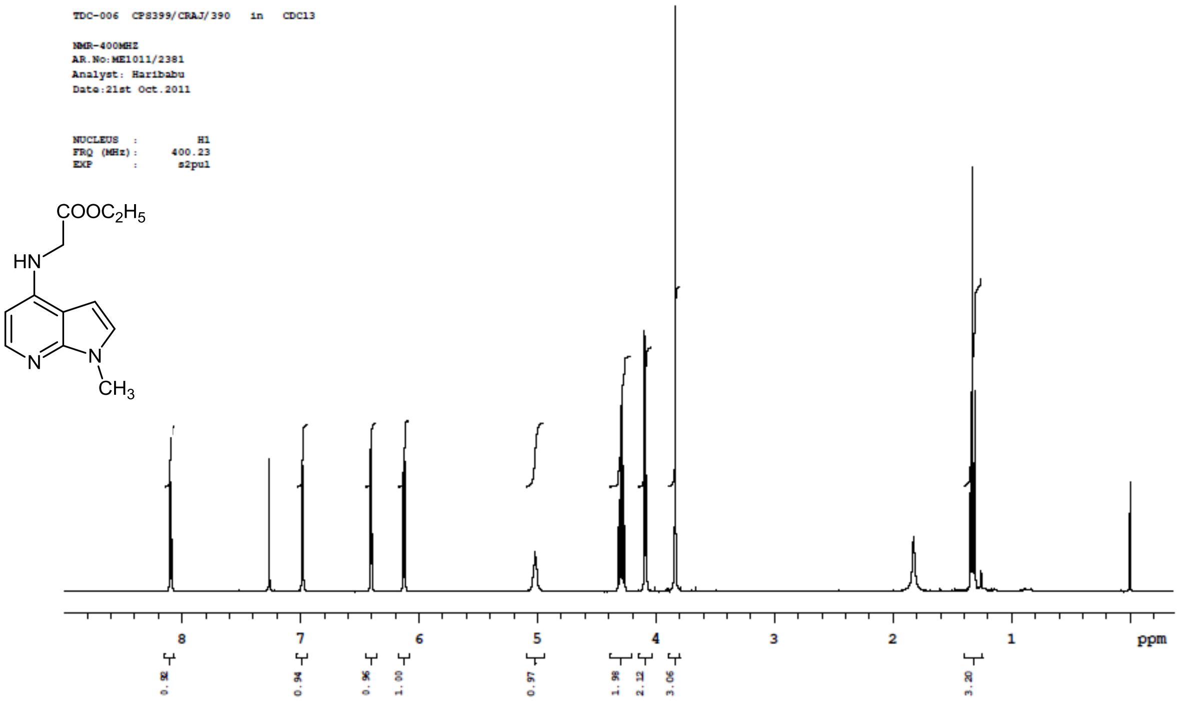

^{13}C NMR of **7c** in DMSO-d_6, 100 MHz.

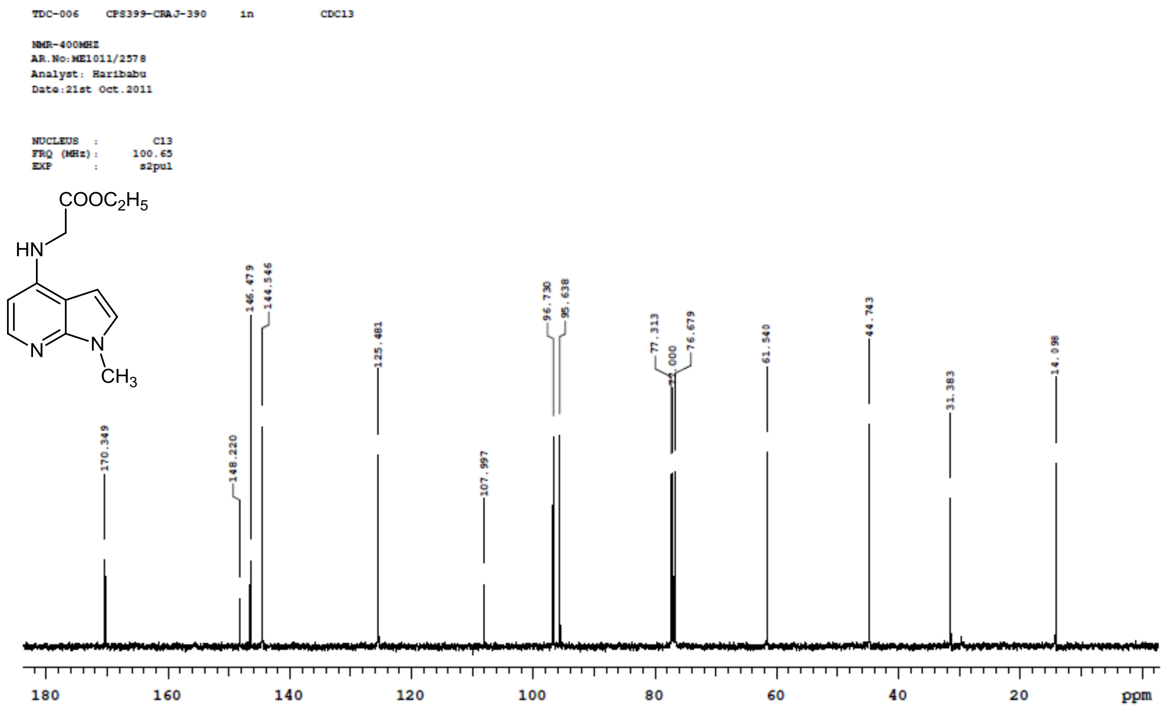

^{1}H NMR of **7d** in DMSO-d_6, 400 MHz.

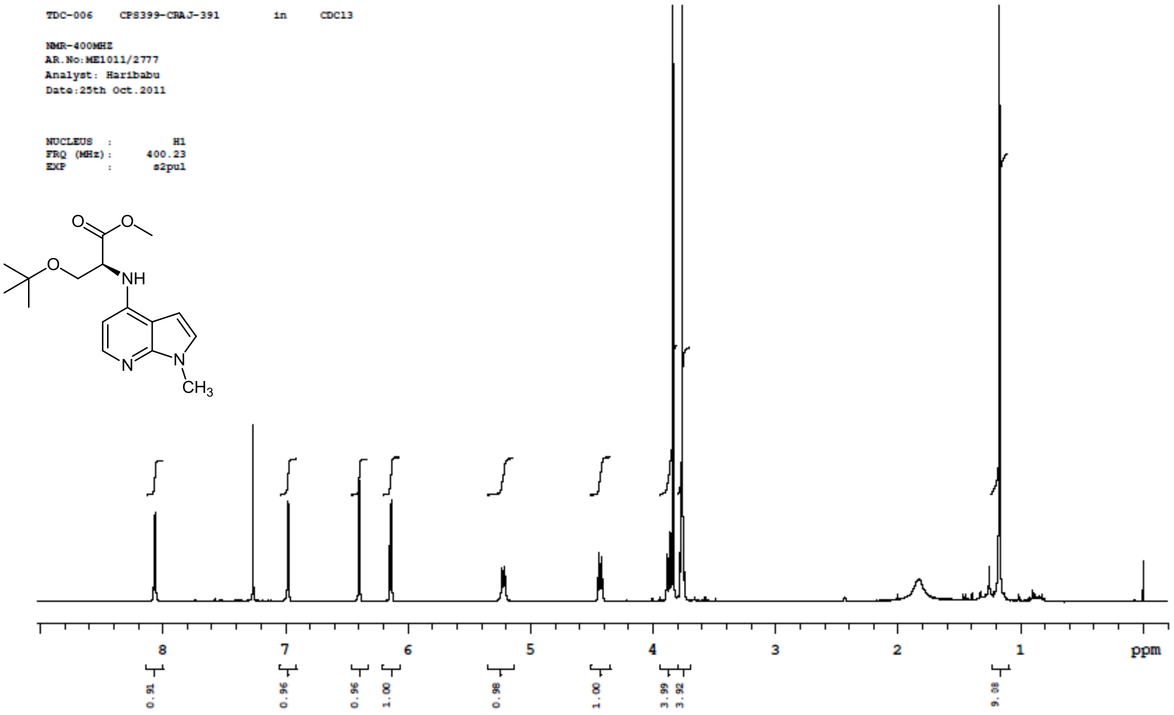

^{13}C NMR of **7d** in DMSO-d_6, 100 MHz.

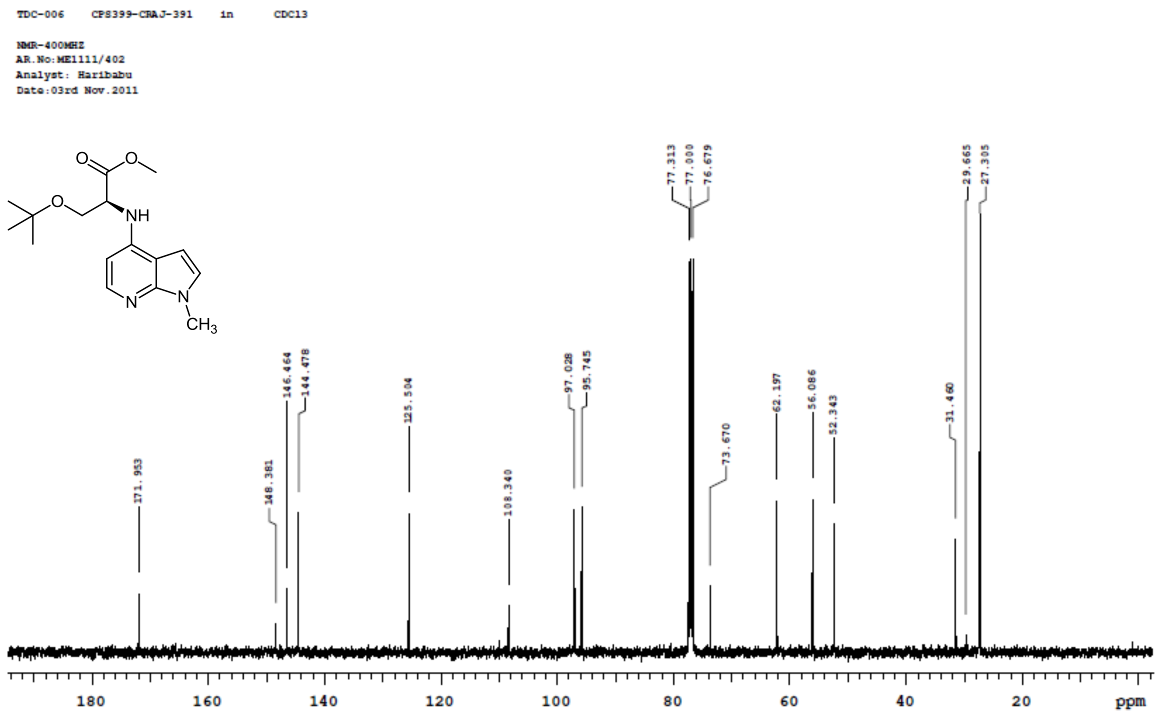

^{1}H NMR of **7e** in $CDCl_3$, 400 MHz.

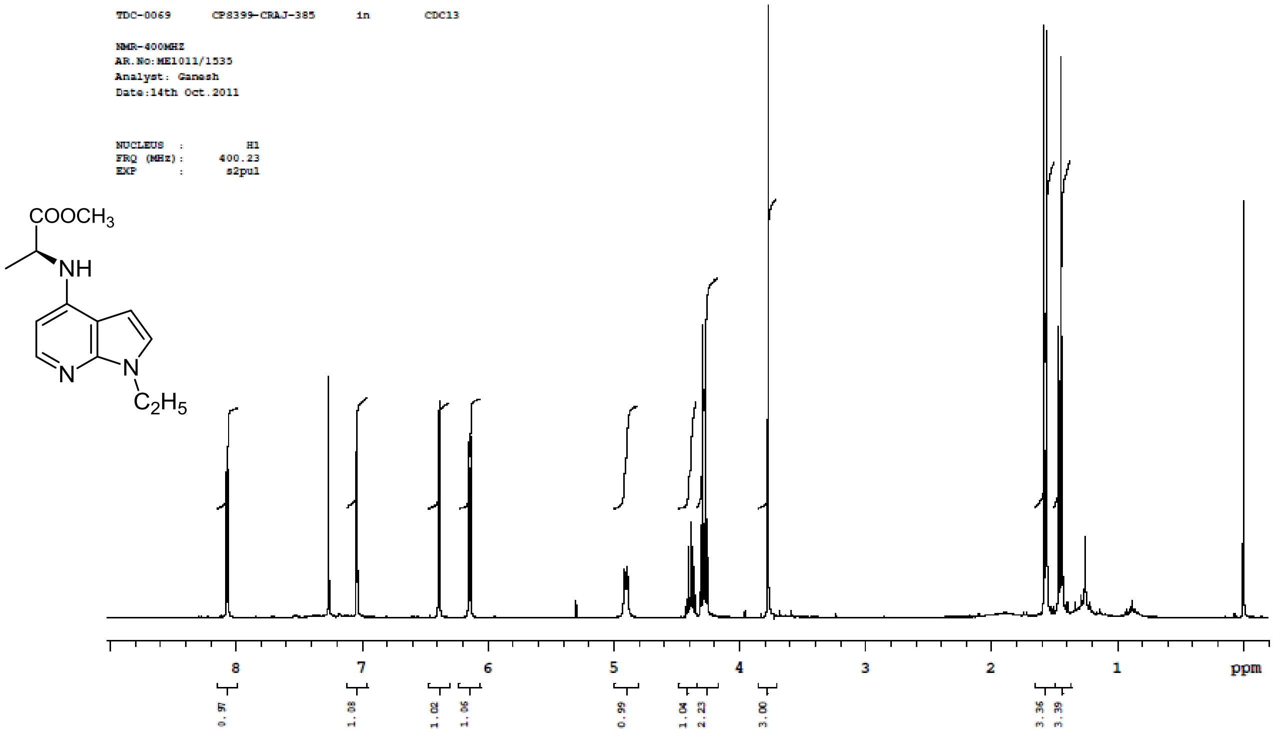

^{13}C NMR of **7e** in $CDCl_3$, 100 MHz.

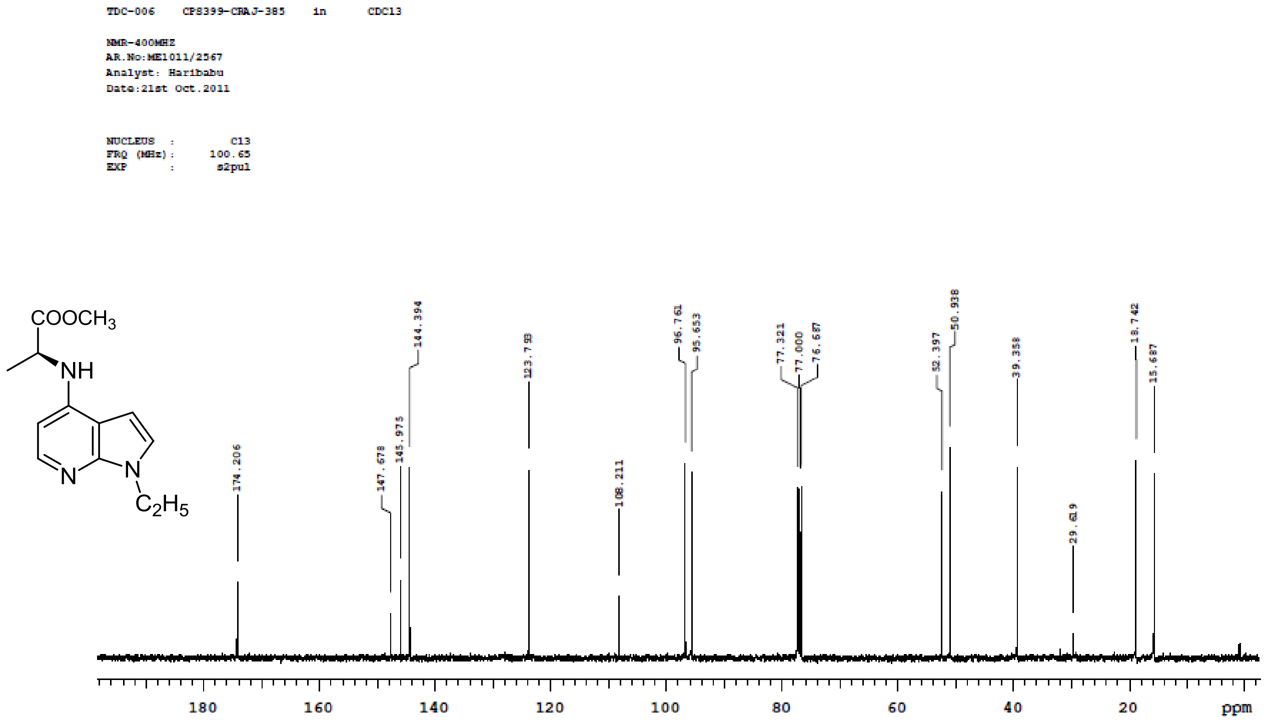

^{1}H NMR of **7f** in $CDCl_3$, 400 MHz.

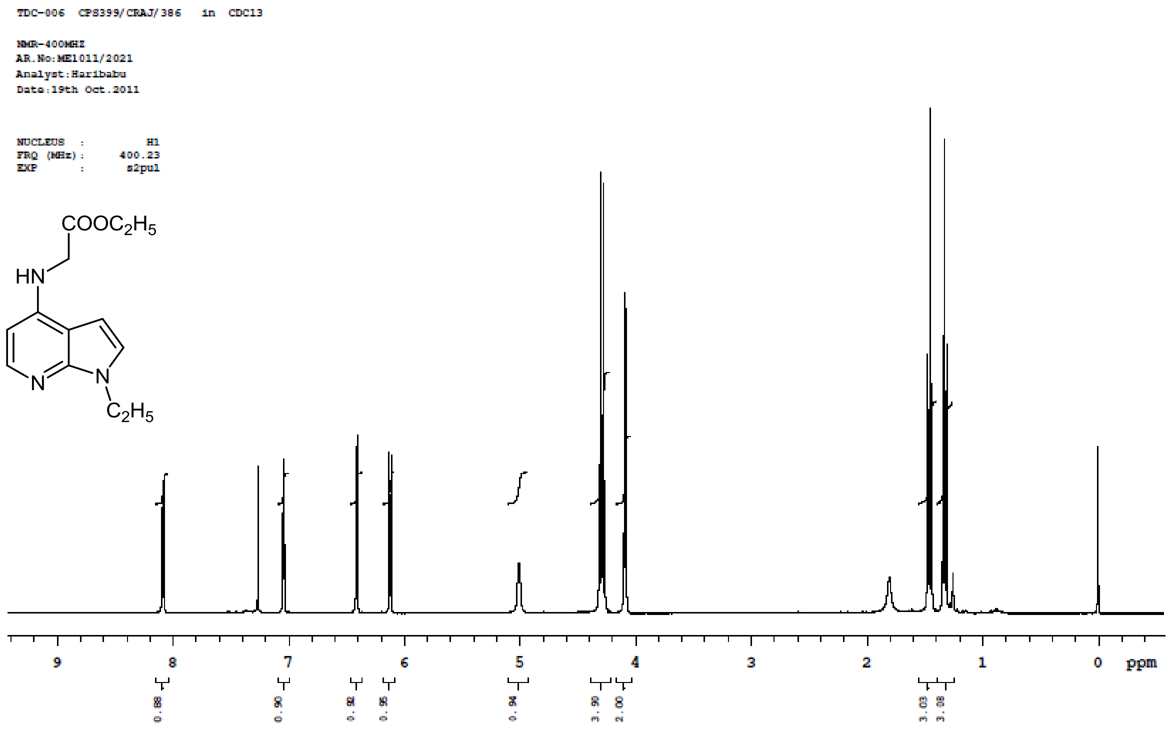

^{13}C NMR of **7f** in $CDCl_3$, 100 MHz.

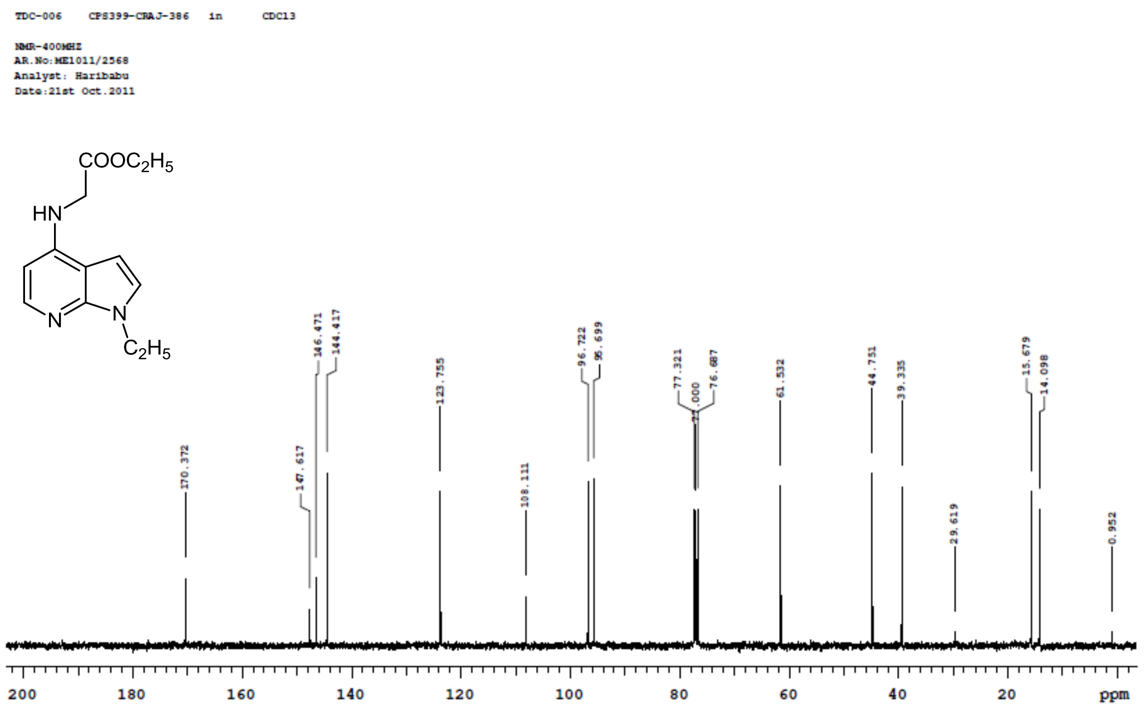

^{1}H NMR of **7g** in $CDCl_3$, 400 MHz.

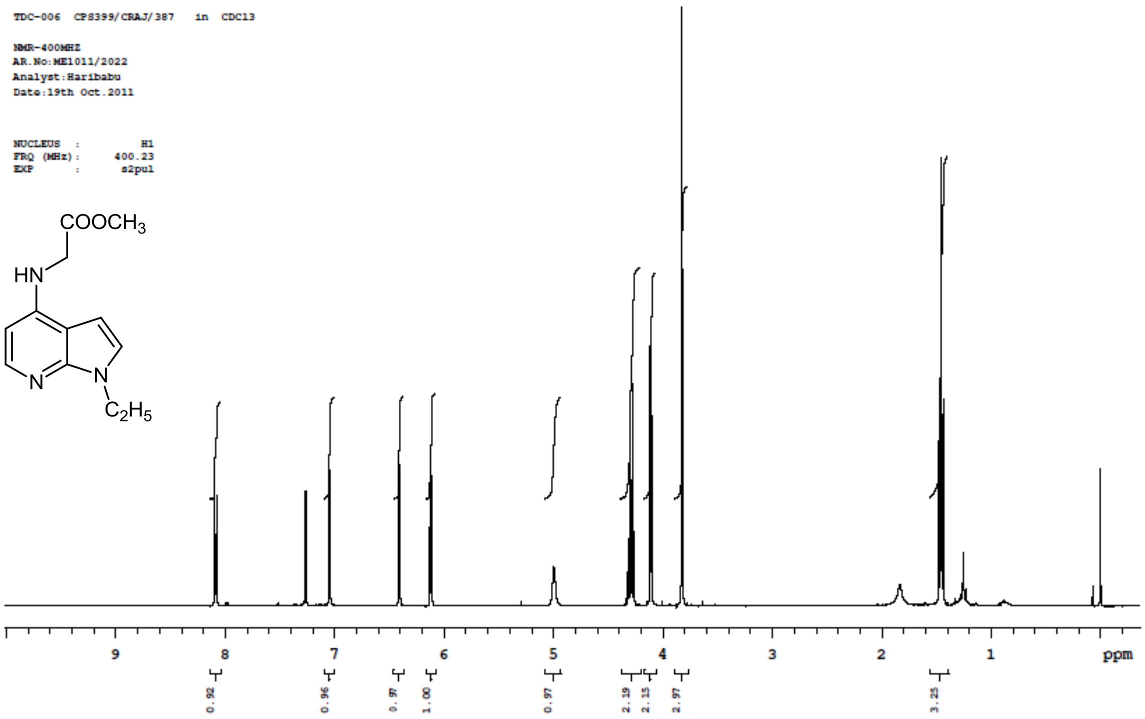

^{13}C NMR of **7g** in $CDCl_3$, 100 MHz.

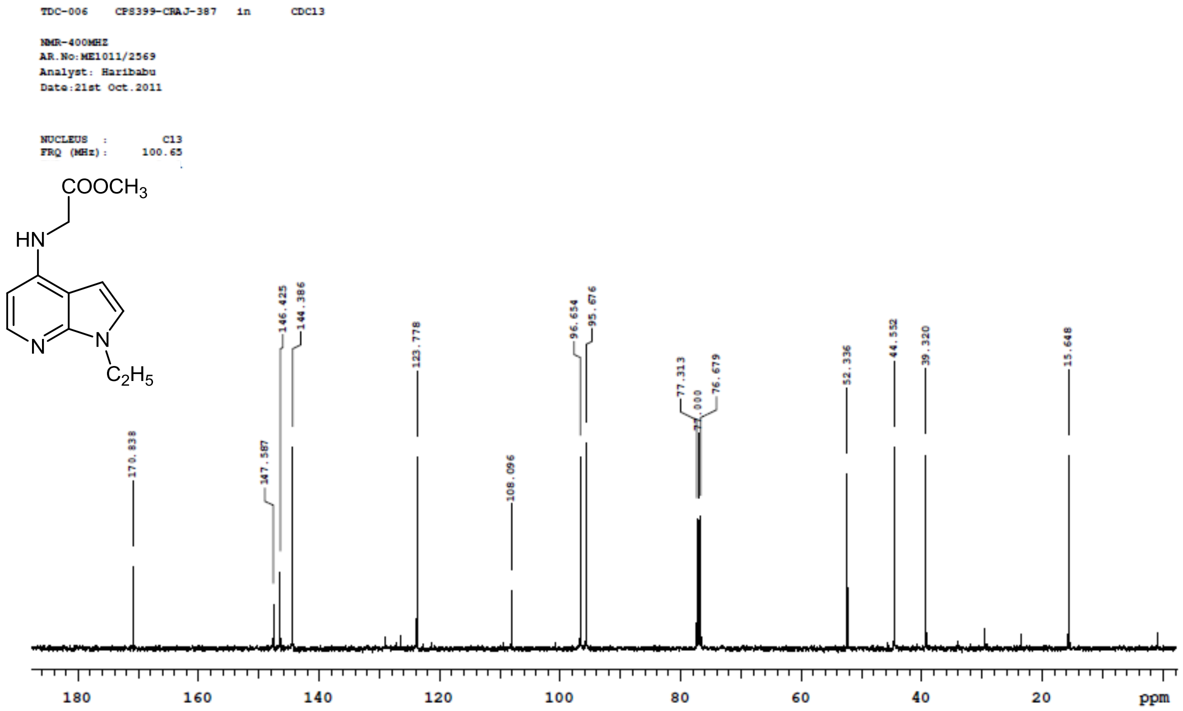

^{1}H NMR of **9a** in $CDCl_3$, 400 MHz.

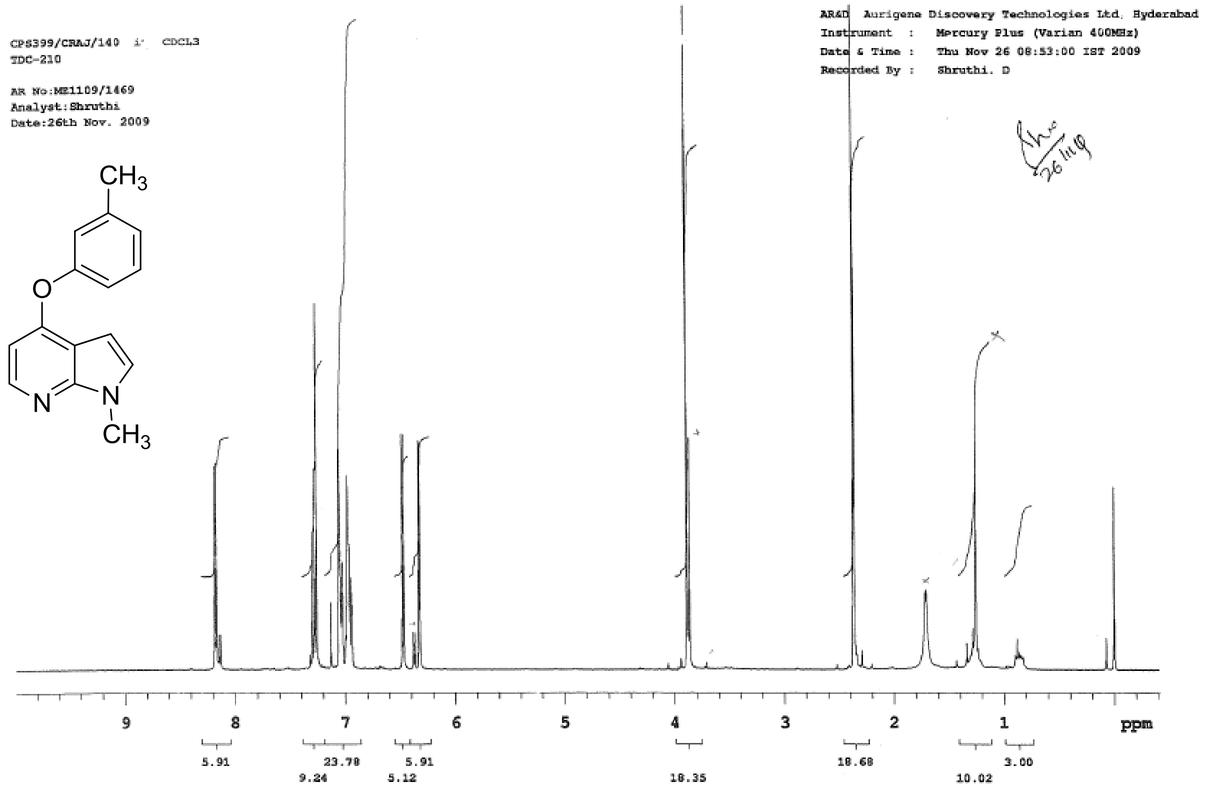

^{13}C NMR of **9a** in $CDCl_3$, 100 MHz.

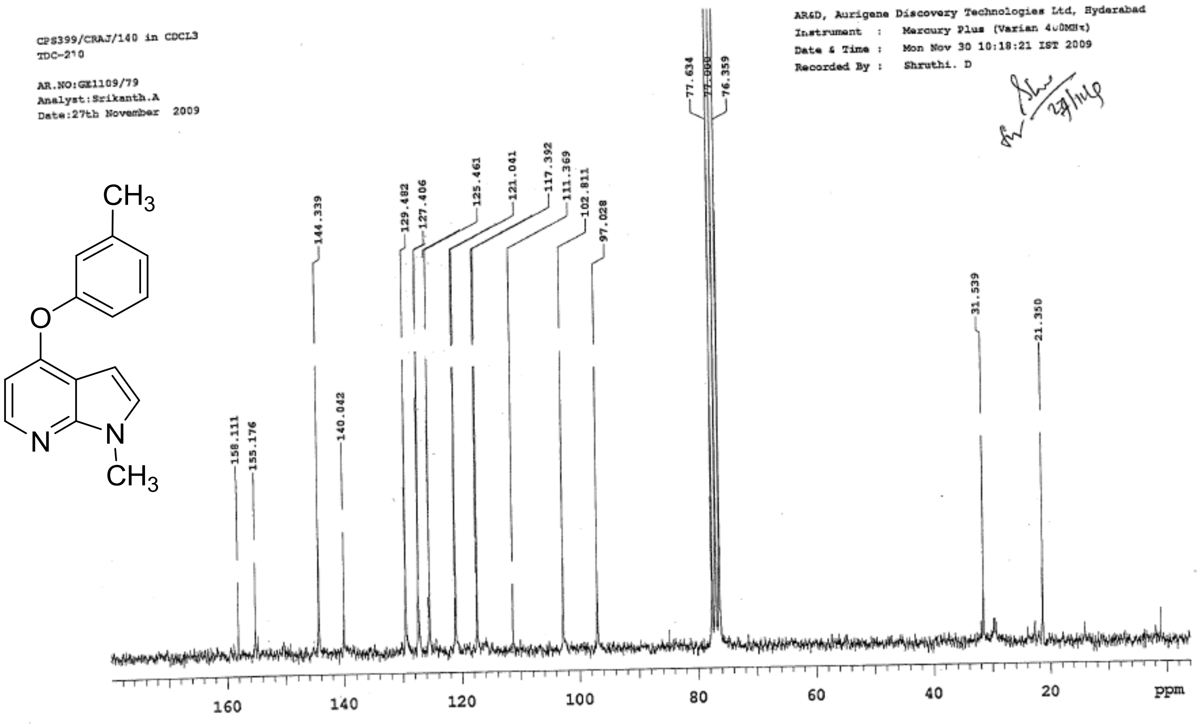

^{1}H NMR of **9b** in $CDCl_3$, 400 MHz.

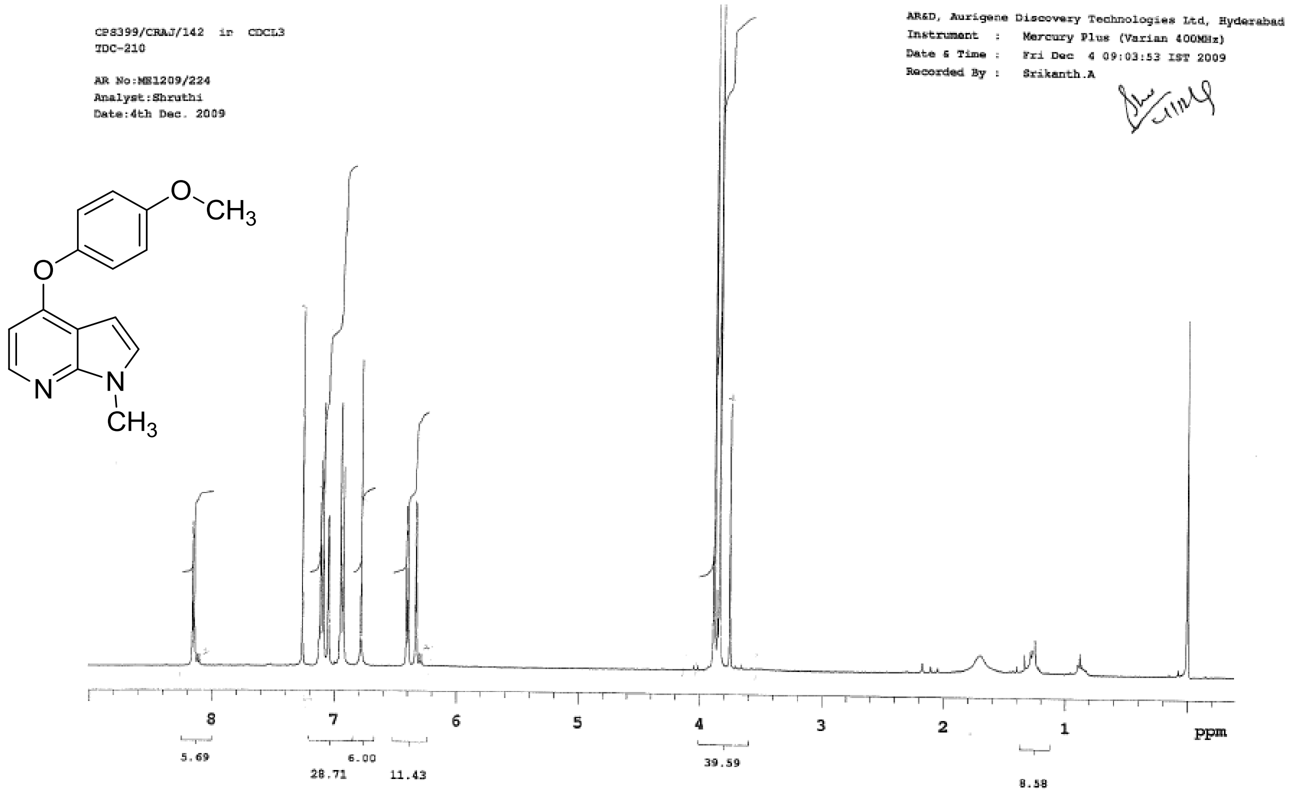

^{13}C NMR of **9b** in $CDCl_3$, 100 MHz.

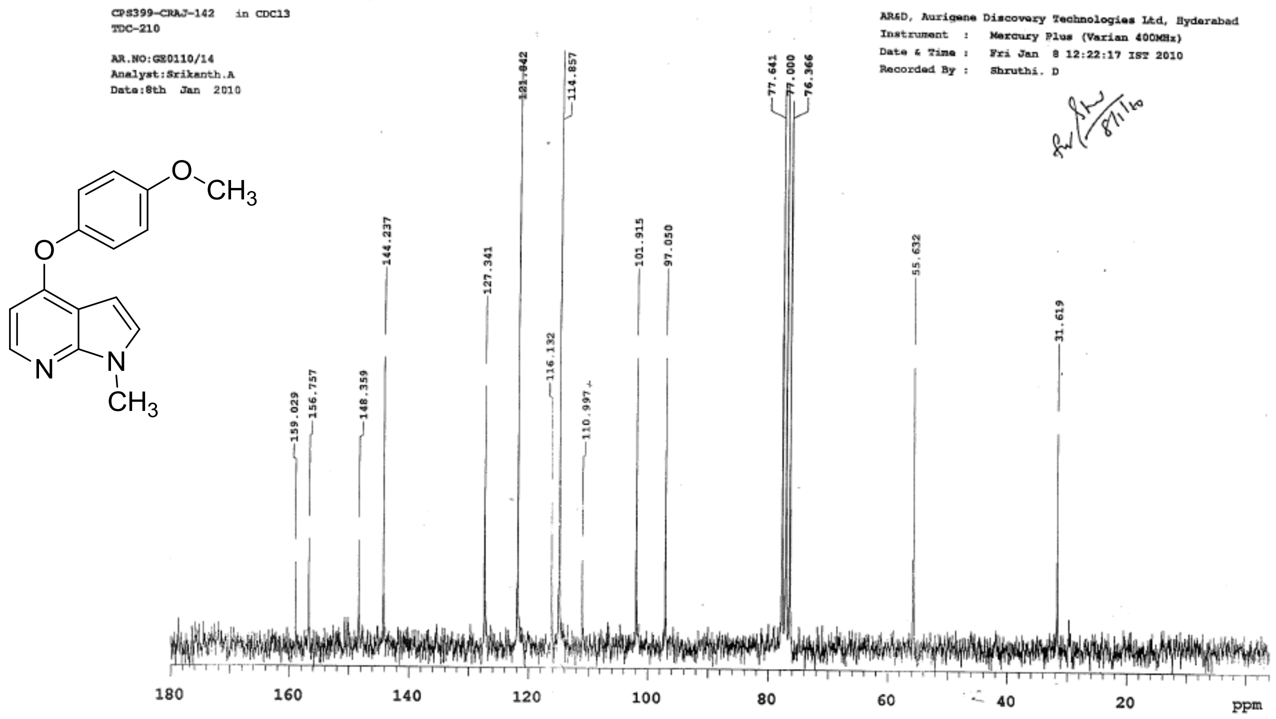

^{1}H NMR of **9c** in $CDCl_3$, 400 MHz.

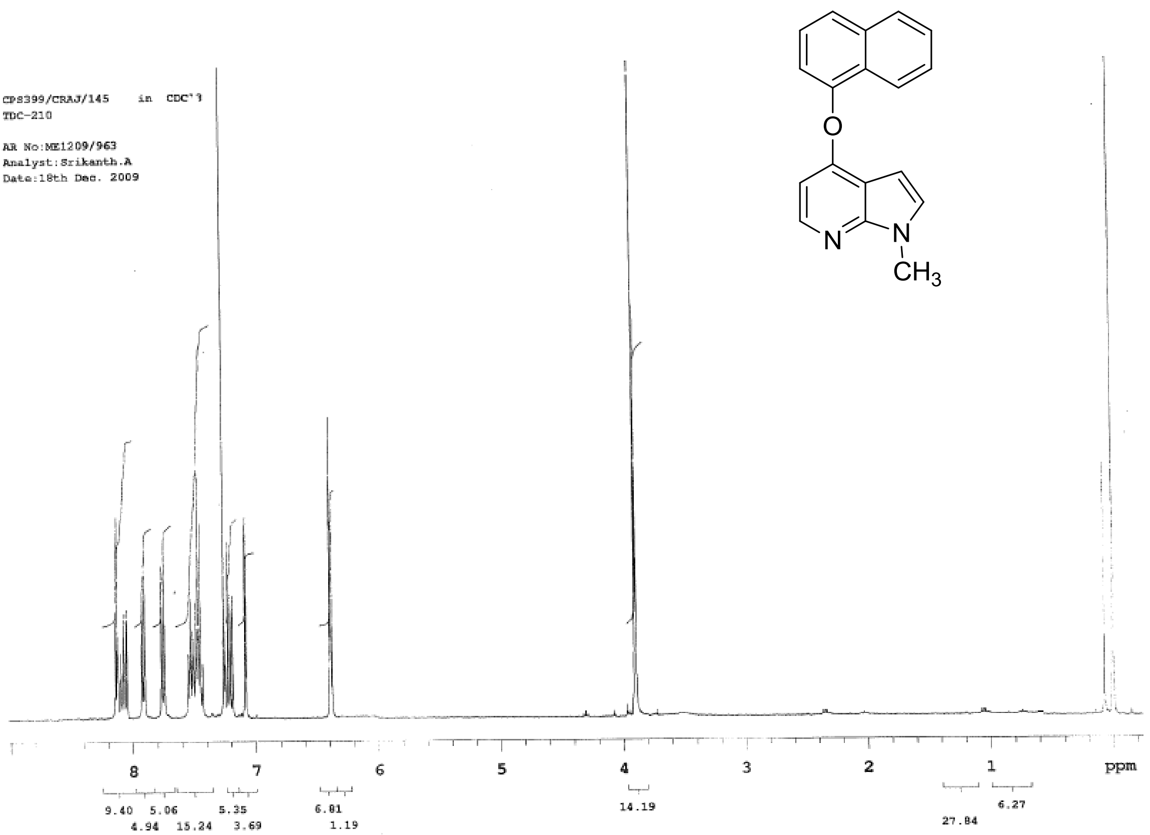

^{13}C NMR of **9c** in $CDCl_3$, 100 MHz.

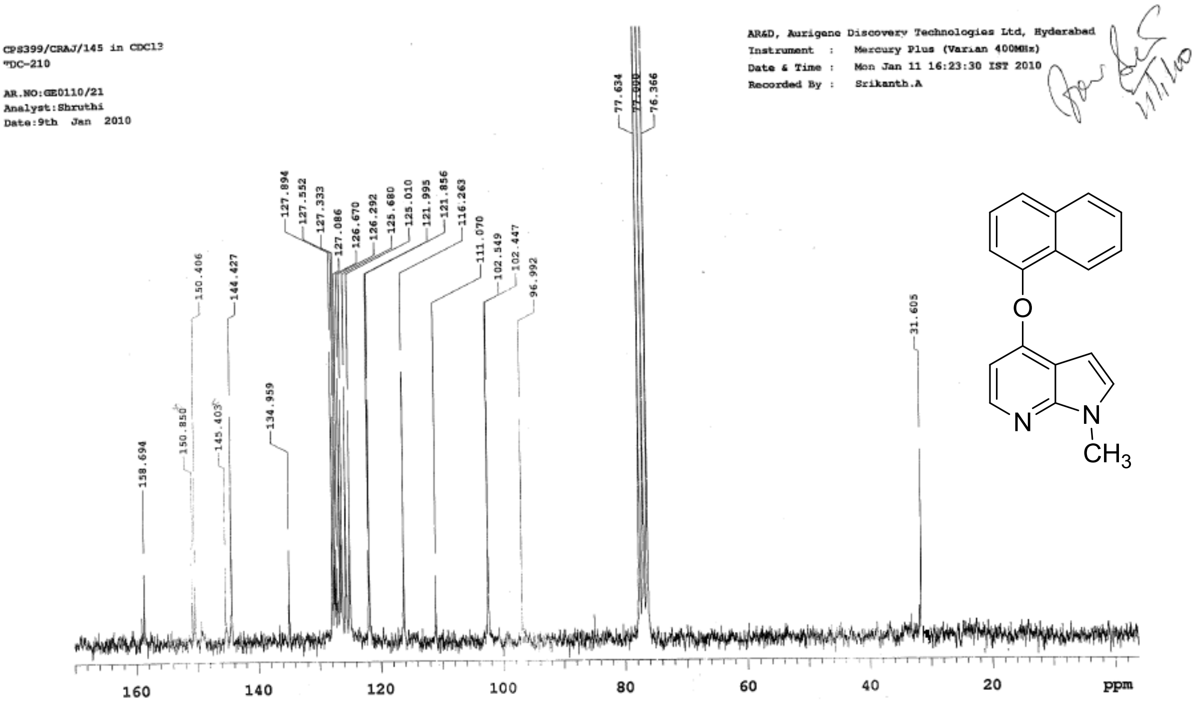

Supplement: File 1 — Experimental procedures, analytical data and NMR spectra. [file Beilstein_J_Org_Chem-08-2004-s001.pdf]
